# Efficacy and Safety of Intralymphatic Immunotherapy With Grass Allergoid and Microcrystalline Tyrosine: A Double‐Blind Randomised Placebo‐Controlled Trial

**DOI:** 10.1111/all.70463

**Published:** 2026-07-29

**Authors:** Lara Šošić, Stephan C. Flory, Claudia C. V. Lang, Emma C. Widmer, Alexandra Chabot, Johannes M. Schmid, Tobias Andermatt, David Melillo, Bjarke Hviid‐Vyff, Maibritt Mardahl, Peter Schmid‐Grendelmeier, Thomas M. Kündig, Hans Jürgen Hoffmann, Pål Johansen

**Affiliations:** ^1^ Department of Dermatology University of Zurich Zurich Switzerland; ^2^ Department of Dermatology University Hospital Zurich Zurich Switzerland; ^3^ Department of Clinical Medicine Aarhus University Aarhus Denmark; ^4^ Department of Respiratory Diseases and Allergy Aarhus University Aarhus Denmark; ^5^ Data‐Set‐Go ApS Silkeborg Denmark

**Keywords:** adjuvant, allergen‐specific immunotherapy, allergic rhinoconjunctivitis, grass pollen allergoid, intralymphatic immunotherapy, microcrystalline tyrosine

## Abstract

**Background:**

Intralymphatic immunotherapy (ILIT) has been investigated for two decades in small clinical trials, typically using native allergens adsorbed to aluminium hydroxide (alum). This randomised, double‐blind, placebo‐controlled study evaluated the safety and efficacy of ILIT with a grass pollen allergoid formulated with microcrystalline tyrosine (MCT).

**Methods:**

Sixty adults with grass pollen–induced allergic rhinoconjunctivitis (ARC) were randomised to receive ILIT with grass allergoid (equivalent to 60 ng Phl p 5; *n* = 31) or placebo (*n* = 29). Three ultrasound‐guided injections (100 μL) were administered into inguinal lymph nodes at 4‐week intervals in early 2022. The primary endpoint was the combined symptom and medication score (cSMS), recorded daily during the 2022 and 2023 grass pollen seasons. Secondary endpoints included safety, Rhinitis Quality of Life Questionnaire (RQLQ), serology, spirometry, fractional exhaled nitric oxide (FeNO), and skin‐prick testing (SPT). An open‐label extension, in which the placebo participants received active ILIT, was conducted in 2024.

**Results:**

ILIT was well tolerated, with no severe adverse events (SAEs). Mild local reactions (wheal, erythema, swelling) occurred after 50 of 93 active injections (53.8%), and three moderate reactions (3.2%) were reported. In the placebo group, one mild and no moderate reactions occurred. No allergen‐specific systemic reactions were observed. Compared with placebo, ILIT reduced cSMS by 33% in 2022 (*p* > 0.05) and 50% in 2023 (*p* < 0.05). RQLQ improved with relative reductions of 22% and 13% (*p* > 0.05), respectively.

**Conclusion:**

ILIT with a grass pollen allergoid formulated with MCT was well tolerated and associated with clinically relevant reductions in symptom and medication use in grass pollen–induced ARC. In this small study cohort, ILIT proved to be efficient and safe, but further optimisation of dosing regimens should follow.

## Graphical abstract

This is the first randomised placebo‐controlled trial of intralymphatic immunotherapy using a microcrystalline tyrosine‐adjuvanted grass pollen allergoid. Three ultrasound‐guided ILIT injections were well tolerated and reduced combined symptom‐medication scores compared with placebo. The findings support further optimisation and clinical development of allergoid‐based ILIT for grass pollen allergy. cSMS, combined symptom medication score; ILIT, intralymphatic immunotherapy; MCT, microcrystalline tyrosine; RQLQ(s), rhinoconjunctivitis quality of life questionnaire (standardised); SPT, skin prick test.

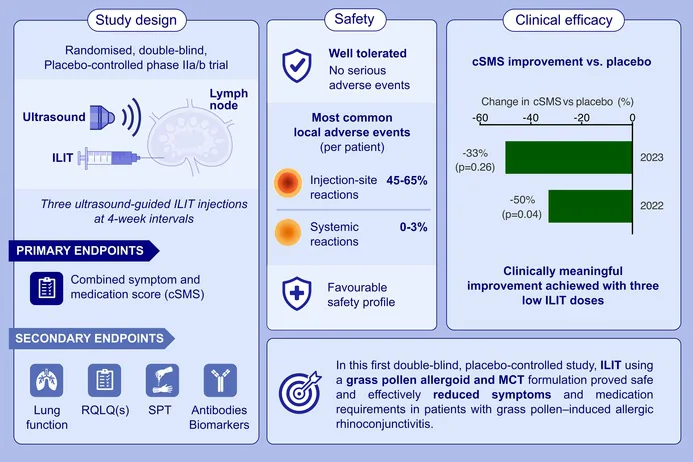

## 1 Introduction

The effectiveness of allergen immunotherapy (AIT) relies on allergen delivery to lymphatic tissue. During subcutaneous immunotherapy (SCIT), less than 5% of the injected allergen reaches regional lymph nodes (due to entrapment and degradation within the subcutaneous tissue) [1]. ILIT directly targets this site by ultrasound‐guided injection of allergen into the lymph node, typically in the inguinal region [2], although axillary lymph nodes have also been used [3]. By delivering allergen to lymph nodes, ILIT avoids cutaneous mast cells and may enhance efficacy while improving safety, reducing dose requirements, and shortening treatment duration, with potential benefits for patient adherence and healthcare costs [4, 5].

Over the past two decades, Phase I and II trials have shown that ILIT induces clinical improvement in allergic rhinoconjunctivitis (ARC) after only three injections [6, 7, 8, 9, 10, 11, 12, 13, 14, 15, 16, 17, 18, 19, 20, 21, 22, 23, 24, 25, 26] Safety and efficacy have been reported to be comparable to those of subcutaneous (SCIT) and sublingual (SLIT) immunotherapy [25, 26, 27, 28]. An injection interval of at least 4 weeks appears appropriate, consistent with vaccination schedules and adequate for immune priming [11, 28, 29].

To date, ILIT studies for grass pollen allergy exclusively used native allergen extract with aluminium hydroxide (alum). No randomised controlled trials have investigated modified allergens (allergoids) or alternative adjuvants in ILIT. Allergoids, chemically or physically modified allergens, typically improve safety while maintaining immunogenicity [30, 31, 32]. Glutaraldehyde treatment polymerises proteins, shielding IgE epitopes, while preserving T‐cell epitopes. Allergoids are well established in SCIT and SLIT, enabling accelerated up‐dosing with favourable tolerability and documented clinical efficacy [33, 34, 35, 36, 37, 38, 39].

In the current Swiss study, we used an allergoid of 13 species of grass with microcrystalline tyrosine (MCT). MCT is a biodegradable and protein‐adsorbing depot adjuvant [40, 41] that resorbs within 1 week [39, 41, 42] and provides controlled allergen release. It has been shown to support affinity maturation and IgG‐induction [43, 44], associated with activation of antigen‐presenting cells and T helper cells without boosting IgE responses [41, 44, 45]. It has demonstrated safety and efficacy in SCIT [39, 46, 47], but has not previously been investigated in ILIT.

Here, we report the first randomised, double‐blind, placebo‐controlled ILIT trial employing either allergoid or MCT, evaluating safety, tolerability, and clinical efficacy in adults with grass‐pollen‐induced ARC. The primary endpoint was the combined symptom medication score (cSMS) during two pollen seasons (2022–2023), with additional clinical and laboratory secondary outcomes, as well as an open label extension prior to the third pollen season (2024).

## 2 Materials and Methods

### 2.1 Trial Design

This phase II double‐blind, placebo‐controlled, randomised controlled trial was conducted in Zurich, Switzerland in 2022–2023. Participants were randomised 1:1 to receive allergen ILIT or placebo (Figure 1A). An independent Data Monitoring Committee reviewed the blinded safety data. One interim efficacy analysis based on cSMS was performed after the first grass pollen season post ILIT (2022). After completion of the double‐blind phase, placebo participants were offered allergen ILIT in 2024 following the same protocol. All participants recorded cSMS and RQLQ during the 2024 season using identical procedures.

**FIGURE 1:**
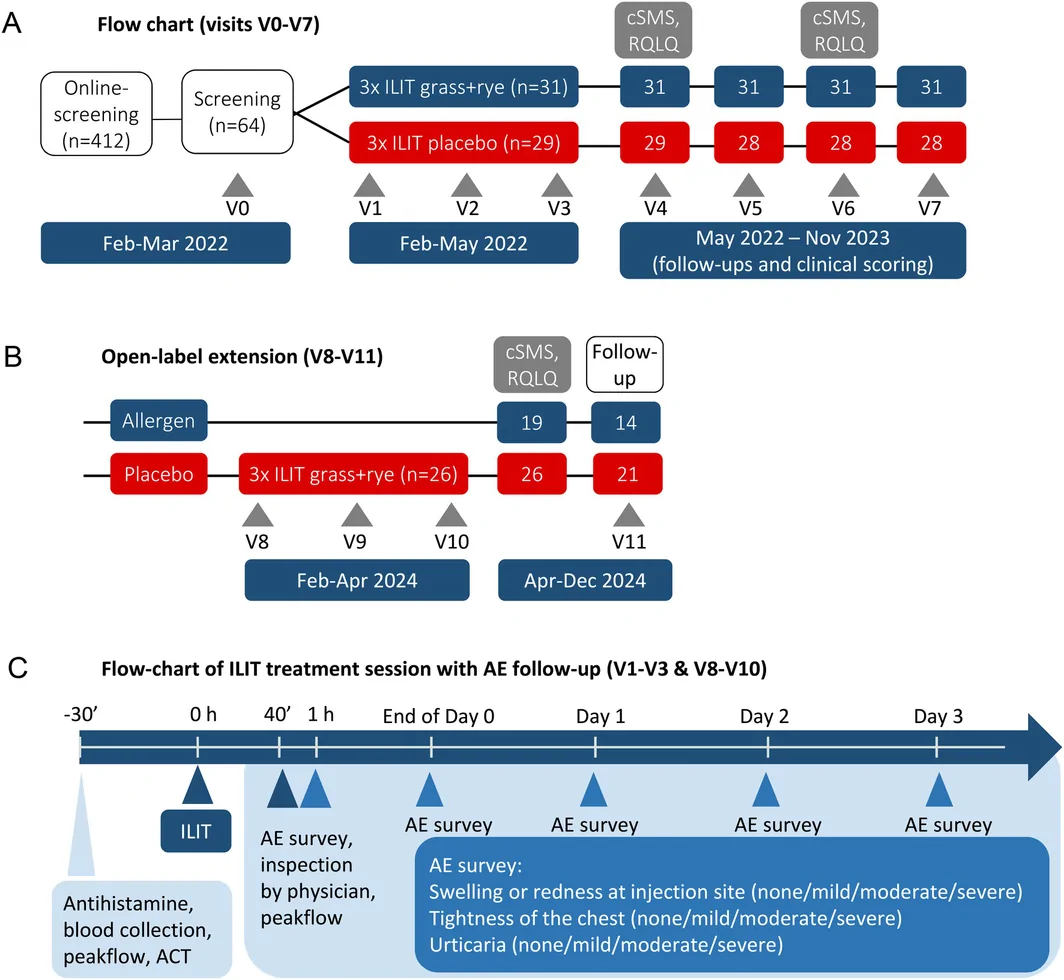
Study participant flow. (A) Initial Trial: Participants were enrolled at visit V0, treated during visits V1–3, and assessed during visits V4–7. Daily cSMS and weekly RQLQ scores were collected during the 3‐month periods around visits V4 and V6. (B) Open‐label extension: Participants initially in the placebo group were offered grass allergoid ILIT during visits V8–10. Follow‐up assessments occurred during the subsequent pollen season and at the final visit (V11). (C) Treatment visits: Each treatment visit (V1–3 and V8–10) included clinical and laboratory assessments, as well as safety monitoring (AE).

### 2.2 Study Participants

Adults aged 18–60 years with grass pollen–induced ARC were eligible. Inclusion criteria were a positive skin prick test (SPT; wheal ≥ 3 mm) to aqueous grass pollen extract and a retrospective Rhinitis Total Symptom Score (RTSS [48]) ≥ 8. Screening included the Respiratory Health in Northern Europe version III (RHINE III) [49]. If asthma was suspected based on RHINE III (Qs 1–6), an Asthma Control Test (ACT) was performed [50].

Exclusion criteria comprised previous AIT for grass pollen, uncontrolled asthma (post‐bronchodilator forced expiratory volume in 1 s (FEV1) < 70% predicted), high‐dose inhaled corticosteroids (ICS, > 800 μg), biologics, relevant concomitant inhalant allergies during grass season, recent anaphylaxis or vaccination, pregnancy, and clinically relevant cardiovascular or immune disorders. The study was conducted between Feb 2022 and Dec 2024 at the Allergy Unit, University Hospital of Zurich.

### 2.3 Intralymphatic Immunotherapy (ILIT) Procedure

Participants received three ultrasound‐guided ILIT injections at 4 to 5‐week intervals (Figure 1A). Each injection consisted of 0.1 mL modified grass allergen (133 Standardised Units (SU), equivalent to 60 ng Phl p 5) or placebo (0.9% saline). Oral desloratadine 5 mg was administered 20–30 min prior to injection.

After groin and probe disinfection, injections were performed under real‐time ultrasound guidance (Acuson X300, Siemens) using an in‐plane technique and recording function [2]. Injection quality was rated using a previously described 1–5 scale [2].

### 2.4 The Investigational Medicinal Product (IMP)

The active product was a standardised for SCIT‐approved grass pollen allergoid of 12 common grass allergens and one rye allergen (Suppl. Text) adsorbed to MCT [51], supplied by Allergy Therapeutics Ltd. (Worthing, UK), and marketed as POLVAC Grasses + Rye in Switzerland by Bencard AG (Greifensee, CH). The formulation contains needle‐like MCT crystals (~20 μm) and 4000 SU/mL allergen. For ILIT, it was diluted threefold in 0.9% saline to 1333 SU/mL. Placebo contained saline only.

### 2.5 Primary Endpoint: Self‐Reporting via Online Questionnaire

The primary endpoint was a ≥ 20% reduction in median cSMS during peak grass pollen season (≥ 4 consecutive days with > 50 pollen grains/m^3^). The cSMS was calculated using the standard method, which combines daily symptom scores (for nasal congestion, sneezing, rhinorrhea, and eye symptoms) with the use of rescue medication (antihistamines, nasal corticosteroids, and corticosteroid tablets), weighted according to a pre‐established scale [52] (Suppl. Text). Participants completed daily online questionnaires via text message links during the 2022 (May 15–August 15) and 2023 (April 15–August 15) seasons (Figure 1A; Suppl. Text).

### 2.6 Secondary Endpoints: Clinical and Serological Measurements

Weekly RQLQ assessments were completed during pollen seasons. SPT was performed at screening and follow‐up with standardised extracts (Allergy Therapeutics Ltd.) including aeroallergen panels (grass mixt, rye, mugwort, birch, alder, ash, hazel, alternaria, house dust mite *D. farinae* and *D. pteronyssinus*, cat, dog, horse) and controls. Wheal diameters were measured as the mean of the longest and perpendicular axes; ≥ 3 mm was considered positive. Spirometry and fractional exhaled nitric oxide (FeNO) were assessed at screening and follow‐up.

Blood samples (total 110 mL per participant) were collected during treatment and follow‐up. Allergen‐specific IgE and IgG4 to timothy grass (g6), rye (g12), Ph p 1 (g205), Phl p 5 (g215), and Phl p 7 + 12 (g214), and total IgE were measured using ImmunoCAP (ThermoFisher Scientific, Waltham, MA, US).

### 2.7 Patient‐Reported Global Symptom Improvement

After the 2022 and 2023 seasons (V5 and V7), participants rated overall symptom change compared to previous seasons (negative = improvement, 0 = unchanged, positive = worsening).

### 2.8 Safety Assessment

Adverse events (AEs) were recorded 40 min post‐injection and via online questionnaires by patient and treating physician for three subsequent days, covering local (swelling, redness), systemic (chest tightness, urticaria), and general symptoms (fatigue, concentration difficulty) (Figure 1C). All AEs were reviewed at follow‐up visits.

### 2.9 Sample Size Calculation

Using G*Power (v3.1), assuming α = 0.05, power 95%, and 10% dropout, 30 participants per group were required to detect a clinically relevant difference in cSMS. One interim analysis preserved overall α = 0.05 (Z = 1.985; *p* = 0.0471).

### 2.10 Randomisation and Blinding

Block randomisation (size six) stratified by gender, age, RTSS, and asthma status was performed using RedCap. Independent pharmacists prepared identical masked syringes to conceal the milky appearance of the active formulation. Needle exchange was performed by an independent nurse. Participants and investigators remained blinded until database lock. The statistician was unblinded for interim analysis.

### 2.11 Statistical Analysis

Analyses were conducted in R (v4.3.1) in the intention‐to‐treat population. Normality was assessed using Shapiro–Wilk testing (α = 0.05). All subject‐level input tables were exported as CSV files. Safety data were summarised descriptively and compared using Fisher's exact test and two‐sided *p*‐values. No adjustment was applied for multiple comparisons, consistent with the exploratory nature of the AE analyses. Immunoglobulins and SPT were analysed as change from baseline. Between‐group comparisons used Mann–Whitney U‐tests and analysis of covariance (ANCOVA). Seasonal treatment effects were analysed using Wilcoxon tests (conf.int. = TRUE) with Hodges–Lehmann estimates and 95% confidence intervals (CI). Analyses were performed on subject‐level medians or means as indicated.

### 2.12 Ethics

The study was approved by the *Cantonal Ethics Committee Zurich* (BASEC 2021–02301), authorised by *Swissmedic* (ID 701220), and registered at ClinicalTrials.gov (NCT05297760). It was conducted according to the Declaration of Helsinki, International Council for Harmonisation Good Clinical Practice (ICH GCP), and Swiss federal Human Research Act and the Clinical Trials Ordinance. All participants provided written informed consent.

## 3 Results

### 3.1 Study Population and Flow

The flowchart in Figure 1A shows that of 412 individuals completing online screening and RTSS assessment, 64 underwent onsite screening (V0). Sixty participants were randomised 1:1 to receive grass allergoid ILIT (*n* = 31) or placebo (*n* = 29). All received three ILIT injections (V1–V3) and attended the first follow‐up (V4). One placebo participant was lost to follow‐up at V5. All 60 participants were included in safety and interim cSMS analyses (interim analysis not shown). Fifty‐nine participants (98%) completed V6–V7 and were included in final analyses under the intention‐to‐treat principle.

Recruitment occurred between February 3 and March 16, 2022, through online advertisement, social media, flyers at the University Hospital of Zurich, and institutional channels. Onsite screening included medical history, examination, spirometry, FeNO, and SPT. Four individuals were excluded (beta‐blocker use *n* = 1; negative SPT *n* = 2; obstructive spirometry *n* = 1). The first participant was enrolled on Feb 7, 2022, and the last on Mar 9, 2022. Treatment ran from Feb 10 to May 17, 2022 (V1–V3), with follow‐up visits in Jun–Jul 2022 (V4), Oct–Nov 2022 (V5), May–Jul 2023 (V6), and Oct–Nov 2023 (V7). Three minor protocol deviations occurred due to scheduling conflicts.

Baseline characteristics are summarised in Table 1. Median age was 29 years (21–59) with 47% females. Ten participants (17%) were smokers and 20 (33%) reported asthma. Median RTSS was 12 (range: 8–18), and median ARIA score was 4 (range: 1–8). Two participants (3%) showed obstructive spirometry (FEV1/FVC < 0.7), and 25 (42%) had FeNO > 25 ppb. In SPT, co‐sensitisation was common. Groups were balanced except for higher house dust mite sensitisation in the ILIT group (58% vs. 10%, *p* < 0.001).

**TABLE 1:** Demographics and baseline characteristics of study population at inclusion. Comparisons between the placebo ILIT and allergen ILIT groups were performed using the Mann–Whitney *U* test for continuous variables and Fisher's exact test for categorical variables.

Characteristic (frequency/range)	Placebo (*N* = 29)	Allergen (*N* = 31)	Total (*N* = 60)
Age in years (median)	28 (21–59)	31 (21–59)	29 (21–59)
Female sex (no.)	14 (48.3%)	14 (45.2%)	28 (46.7%)
Race group (no.)			
White	28 (96.6%)	30 (96.8%)	58 (96.7%)
Asian	1 (3.4%)	1 (3.2%)	2 (3.3%)
Body‐mass index (kg/m^2^, median)	24 (17–35)	23 (18–31)	23.5 (17–35)
Current smoker (no.)	3 (10.3%)	7 (22.6%)	10 (16.7%)
History of asthma (no.)	8 (27.6%)	12 (38.7%)	20 (33.3%)
RTSS (median)	13 (8–18)	12 (6–10)	12 (8–18)
ARIA Questionnaire (median)	4 (1–8)	4 (1–8)	4 (1–8)
SPT diameter (mm, median)			
Histamine	5.0 (3.0–11.5)	5.0 (3.0–11.8)	5.0 (3.0–11.8)
Grass	8.5 (4.5–12.5)	7.5 (4.0–12.5)	8.0 (4.0–12.8)
Rye	2.3 (0.0–16.8)	9.0 (4.0–21.0)	2.1 (0.0–21.0)
IgE median baseline (kUA/L, range)			
Timothy grass	15.7 (2.1–79.9)	14.3 (0.7–130.8)	15.4 (0.7–130.8)
Rye	11.9 (1.4–58.5)	9.1 (0.5–69.7)	11.8 (0.5–69.7)
Phl p 1	9.2 (1–36)	10.2 (0.3–50.4)	9.7 (0.3–50.4)
Phl p 5	7.6 (0–49.5)	6.6 (0–100.1)	6.7 (0–100.1)
Phl p 7 + *p* 12	0.1 (0–14.1)	0 (0–3.1)	0 (0–14.1)
Total IgE median baseline (kU/L)	101 (19–574)	147 (21–1365)	122 (21–1365)
IgG4 median baseline (kUA/L, range)			
Timothy grass	0.5 (0.1–2)	0.3 (0.1–2.8)	0.3 (0.1–2.8)
Rye	0.2 (0–1.4)	0.1 (0–2.1)	0.1 (0–2.1)
Phl p 1	0 (0–0.4)	0 (0–4.8)	0 (0–4.8)
Phl p 5	0 (0–0.6)	0 (0–4)	0 (0–4)
Phl p 7 + *p* 12	0 (0–1.5)	0 (0–2.7)	0 (0–2.7)
Co‐sensitisations, SPT (total no.)	4 (0–8)	5 (0–10)	5 (0–10)
Mugwort pollen (positives)	8 (27.6%)	8 (25.8%)	16 (26.7%)
Mould spores (positives)	1 (3.4%)	3 (9.7%)	4 (6.7%)
Tree pollen (positives)	22 (75.8%)	21 (67.7%)	43 (71.7%)
House dust mites (positives)	3 (10.3%)	18 (58.1%)	21 (35.0%)
Animal dander (positives)	13 (44.8%)	18 (58.1%)	31 (51.7%)
Lung function (median)			
FEV1 (litres)	4.09 (2.7–5.5)	3.79 (2.5–5.7)	3.84 (2.5–5.7)
% of predicted value	100 (79–116)	100 (79–116)	97 (75–130)
FVC (litres)	4.80 (3.3–8.4)	4.48 (2.7–7.3)	4.62 (2.7–7.2)
% of predicted value	97 (78–126)	98 (60–138)	97 (60–138)
FEV1/FVC	0.82 (0.74–0.96)	0.80 (0.62–1.00)	0.81 (0.62–1.00)
FeNO (ppb)	12 (6–72)	20 (6–72)	19 (6–72)

During the 2024 open‐label extension (Figure 1B), 26/29 placebo participants received active ILIT; 19/31 originally treated participants also completed cSMS and RQLQ questionnaires in 2024.

### 3.2 Safety and Tolerability

No SAEs occurred and ILIT was well tolerated (Table 2). The ultrasound‐guided procedure required approximately 5–10 min and was not associated with pain, but some patients reported a transient pulling sensation during injection (not systematically recorded); the post‐ILIT safety monitoring scheme is shown in Figure 1C. Across 180 injections, 63% and 66% caused no AEs during the 40‐min observation period, as recorded by the treating physician and participants, respectively (Table S1). Mild local reactions occurred after 30% or 33% of injections, predominantly in the ILIT group (53 vs. 1; *p* < 0.001, Table S2). The cumulative numbers of reported local AEs were 43 for the five monitoring timepoints (40 min, day 0, day 1, day 2, and day 3 post injection) after the first allergoid ILIT, and the numbers increased to 58 for the following two ILIT sessions (Figure 2). Three moderate local reactions were confirmed (Table S2). Generalised urticaria (*n* = 2) and chest tightness (*n* = 5; 4 confirmed) were infrequent and similar between groups. Of note, the duration of each AE varied between individuals, meaning that the cumulative number of AEs at a given visit may exceed the number of patients experiencing the same AE. No respiratory compromise was observed.

**TABLE 2:** Numbers and frequencies of AEs as reported by physician and study participant during the 3 days of safety monitoring following all three ILIT injections. The analyses were conducted per patient and in participants with 1 or more AEs per monitoring timepoint. No imputation of missing data was performed.

Symptom, time after ILIT	Placebo ILIT	Allergen ILIT	Fisher *p*
Injection‐site swelling/redness			
40 min (physician)	1/29 (3.4%)	25/31 (80.6%)	< 0.01
Day 1	3/29 (10.3%)	19/31 (61.2%)	< 0.01
Day 2	2/29 (6.9%)	12/31 (38.7%)	0.01
Day 3	0/29 (0.0%)	6/31 (19.3%)	0.02
Chest pressure/difficulty breathing			
40 min (physician)	2/29 (6.9%)	1/31 (3.2%)	0.61
Day 1	3/29 (10.3%)	0/31 (0.0%)	0.11
Day 2	1/29 (3.4%)	0/31 (0.0%)	0.48
Day 3	1/29 (3.4%)	2/31 (6.4%)	1.00
Generalised urticaria			
40 min (physician)	0/29 (0.0%)	0/31 (0.0%)	1.00
Day 1	1/29 (3.4%)	3/31 (9.6%)	0.61
Day 2	2/29 (6.9%)	2/31 (6.4%)	1.00
Day 3	2/29 (6.9%)	1/31 (3.2%)	0.61
Difficulty concentrating			
Day 1	6/29 (20.6%)	4/31 (12.9%)	0.50
Day 2	2/29 (6.9%)	2/31 (6.4%)	1.00
Day 3	4/29 (13.7%)	1/31 (3.2%)	0.19
Fatigue			
Day 1	16/29 (55.1%)	14/31 (45.1%)	0.61
Day 2	12/29 (41.3%)	8/31 (25.8%)	0.27
Day 3	10/29 (34.4%)	7/31 (22.5%)	0.39

**FIGURE 2:**
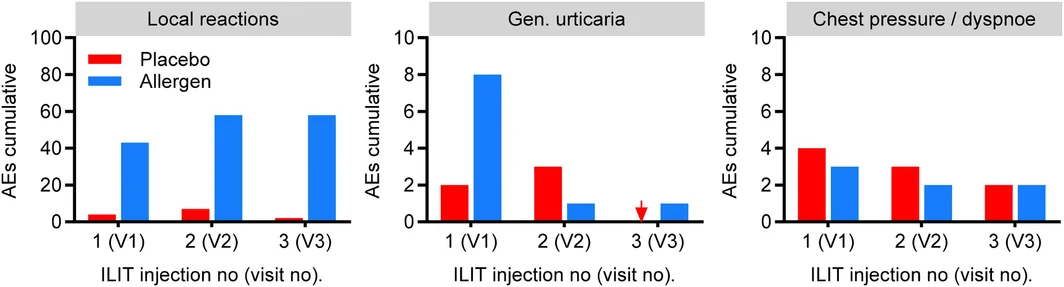
Safety assessment during the treatment phase. Cumulative patient reported adverse events (AEs) during the 3‐monitoring period (40 min post‐injection, Day 0, Day 1, Day 2, and Day 3) are shown for the injection site reactions (*left panel*), generalised urticaria (*middle panel*), and chest pressure or dyspnoea (*right panel*) during the initial randomised, double‐blind treatment phase (V1–V3). The bars represent the total number of AEs reported, categorised by allergen or placebo ILIT. Note that each participant could report a single AE up to five times, resulting in cumulative totals per patient; the urticaria score of 8 after the first allergen ILIT reflects repeated reporting by 3 participants. The analysis was performed per patient.

Within the first day after discharge from the allergy ward, the number of participants without any AEs increased from 114 (63%) to 139 (77%). The local reactions maintained in 51 cases (28%), none of moderate degree. Mild generalised urticaria was reported in two (1%) cases by participants, one of them with known chronic spontaneous urticaria. There were four (2%) reports of mild chest tightness.

Delayed local reactions declined over the 3 days following injection, with persistent local reactions reported in only 7 (4%) cases on day 3. Generalised urticaria or chest tightness was reported sporadically (1%–2%) on days 1–3, primarily after the first ILIT injection and equally associated with allergoid and placebo ILIT. All AEs resolved with oral antihistamines. No treatment was required for chest tightness, no respiratory compromise was documented, and no SAEs occurred.

### 3.3 Quality of Intralymphatic Injection

The mean injection quality score was 4.7 (SD = 0.6) (data not shown). Fifty‐two study participants (86.7%) received three successful injections into the lymph node (hilus or cortex; scores 4–5), seven participants (11.7%) received two successful injections, and one (1.7%) received a single successful injection [2].

### 3.4 Patient‐Reported Outcomes: cSMS and RQLQ

Response rates for daily cSMS were 90.0% (allergoid ILT, 5970 daily entries) and 85.6% (placebo ILIT, 5314 entries), and for weekly RQLQ, the response rates were 85.1% (791 entries) and 78.9% (686 entries), respectively (Figure 3A). All participants achieved > 60% response completeness for both questionnaires.

**FIGURE 3:**
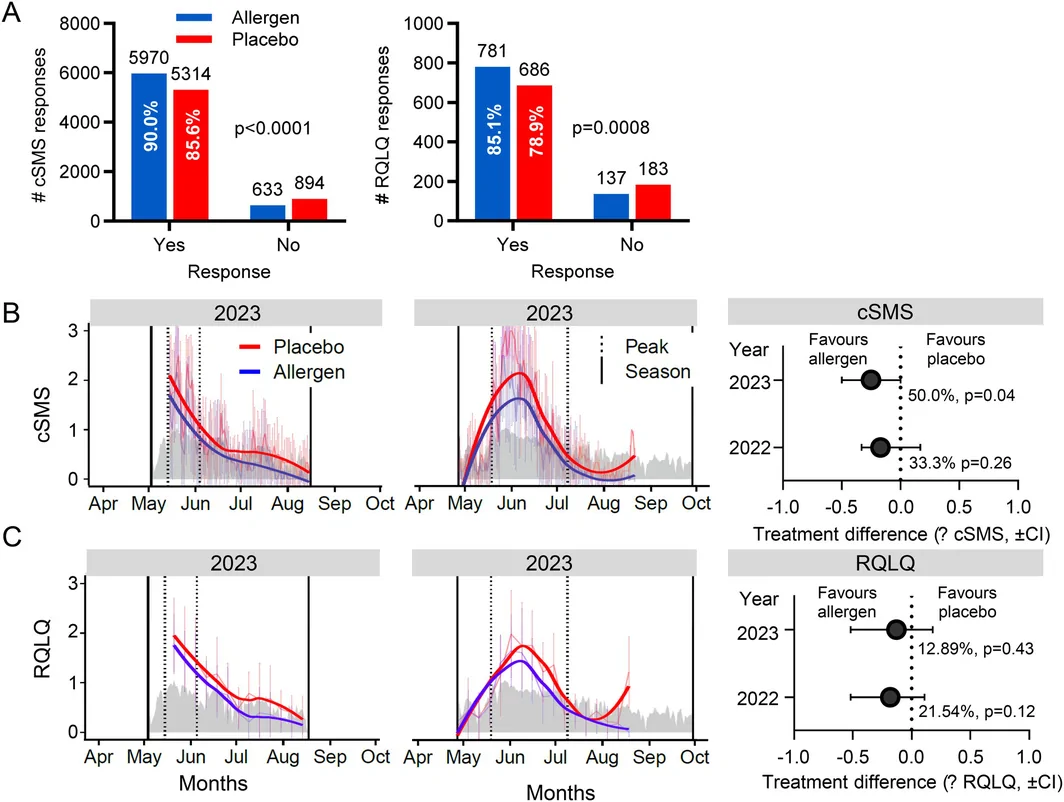
Seasonal monitoring of symptoms, medication usage, and quality of life. (A) Response rates for daily cSMS (left) and weekly RQLQ (right). (B) cSMS and (C) RQLQ reporting during the first (15 May–15 Aug 2022) and second (15 Apr–15 Aug 2023) pollen seasons after ILIT with allergen (*blue*) or placebo (*red*). Smoothed cSMS and RQLQ with 95% CI plotted against log‐scaled daily pollen counts (grains/m^3^, grey area) over time for 2022 and 2023. Pollen seasons are indicated by solid vertical lines, peak periods by hatched lines. Treatment effect analyses for cSMS (Wilcoxon rank‐sum test) and RQLQ (Welch t‐test) were based on subject‐level aggregates during peak pollen seasons.

The pollen exposure varied markedly between seasons, with pollen peak load being 2596 grains/m^3^ in 2022, 6326 in 2023, and only 585 in 2024 (Figure S1A,B). Moreover, while the grass pollen seasons for 2022, 2023, and 2024 were 105, 154, and 128 days respectively, the peak pollen seasons, periods with at least 4 consecutive days with > 50 pollen grains/m^3^, were 29, 50 and 12 days, respectively (Figure S1C). Figure 3B illustrates the seasonal cSMS profiles alongside regional pollen counts. As the 2022 reporting window (15 May–15 Aug) opened later than the onset of the pollen season, the 2023 windows were advanced to start on 15 April. Therefore, the effect analysis for 2022 was missing several days expected to be included in the early season.

Compared with placebo, ILIT reduced cSMS by 33.3% in 2022 (*p* = 0.26) and 50.0% in 2023 (*p* = 0.04), corresponding to mean reductions of 0.17 and 0.25 points (Figure 3B; Table S3). In an additional mixed‐model sensitivity analysis using mean cSMS values, the treatment effect was more pronounced at higher pollen exposure (> 130 grains/m^3^), with effect sizes of 18.4% in 2022 (*p* = 0.13) and 21.5% in 2023 (*p* = 0.04), compared with 18.7% (*p* = 0.17) and 19.4% (*p* = 0.08) when all data were included (Figure S2). Interestingly, the benefit of ILIT on the cSMS was more strongly related to the effect on symptoms (2022: 26.0%, *p* = 0.08; 2023: 26.4%, *p* = 0.04) than to medication (Figure S3).

After treatment of the placebo group in the Open Label Extension in 2024, no between‐group difference was registered (−11.11%, *p* = 0.59; Figure S4A and Table S3). RQLQ analysis showed similar trends to the cSMS results, with effect sizes of 21.5% in 2022 and 12.9% in 2023 (Figures 3C, S4B and Table S4), but the effect sizes were not statistically significant.

When asked about the overall symptom change compared to previous seasons, participants reported greater subjective improvement with ILIT after both seasons, the differences being 27.1% (*p* = 0.017) in 2022 and 29.9% (*p* = 0.123) in 2023 (Figure S5).

### 3.5 SPT, Spirometry, and FeNO Testing

No between‐group differences were observed for SPT to grass or rye pollen (Figure 4A, Table S5). A statistically significant difference between placebo and ILIT in adjusted mean change from baseline was observed for FEV1% pred at the autumn follow‐up visits in 2022 (V5) and 2023 (V7) (*p* < 0.05), and compared with baseline, FEV1% pred increased significantly in the ILIT group (*p* < 0.05) (Figure 4B, Table S6). No consistent differences were determined for the spirometric parameters FVC or the FEV1/FVC ratio.

**FIGURE 4:**
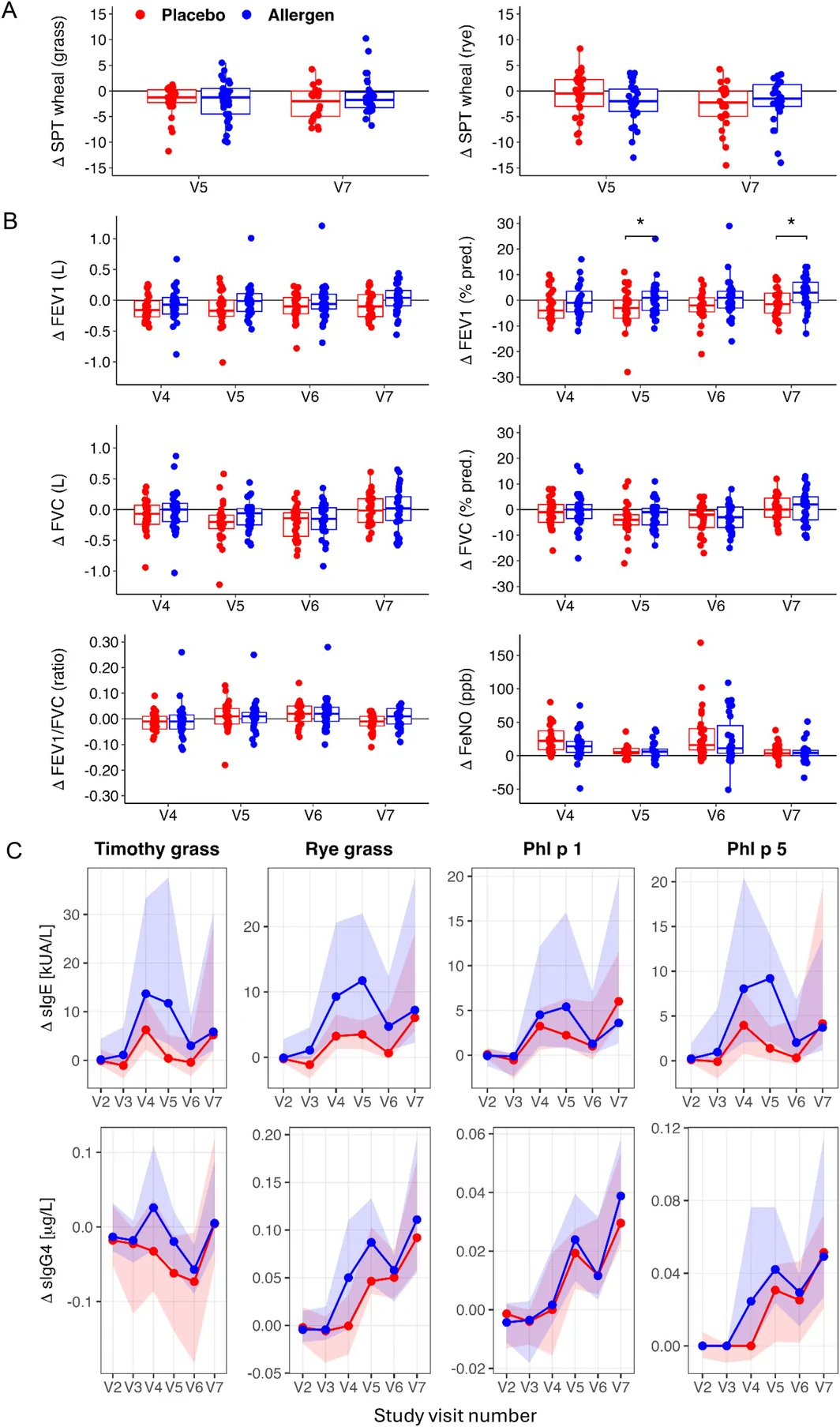
Effect of ILIT on the secondary outcomes. (A) Changes in skin‐prick test (SPT) reactivity to grass and rye pollen at winter visits V5 and V7, expressed as change in wheal diameter relative to baseline (V0). (B) Lung function and airway inflammation outcomes, including changes in FEV1, FVC, FEV1/FVC ratio, and FeNO, assessed at visits V4–7 and expressed as change from baseline (V0). FEV1% pred and FVC% pred denote values expressed as percentages of predicted normal values. Boxplots show the 25th percentile, median, and 75th percentile; dots represent individual participants. Adjusted means and significance testing were derived using ANCOVA models. *p* < 0.05. (C) Changes (Δ) in allergen‐specific IgE (*top*) and IgG4 (*bottom*) to timothy and rye grass extracts, as well as the major timothy grass components Phl p 1 and Phl p 5, relative to baseline (V0). Values with confidence intervals are shown for visits V2–7. Blue symbols and lines indicate allergen ILIT and red indicates placebo ILIT.

FeNO increased with pollen exposure in placebo participants but was attenuated in the ILIT group at V7 (data now shown). ILIT significantly reduced pollen‐associated FeNO rises in 2022 (interaction *p* = 0.002), with a similar trend in 2023 (Figure S6, Table S7). At the mean pollen counts of each follow‐up period, allergen ILIT reduced FeNO versus placebo by 19.8 ppb (*p* = 0.001) in 2022 and 13.8 ppb (*p* = 0.126) in 2023, highlighting stronger anti‐inflammatory effects under higher pollen exposure.

In the non‐asthmatic patients, a significant increase of FEV1% pred was determined in the allergoid ILIT group compared to placebo at visits V4–7. Similarly, in the non‐asthmatic subgroup, a decrease in FeNO was determined at V4. In contrast, the asthmatic subgroup did not show any significant change in FEV1% pred or FeNO following treatment (Figure S1).

### 3.6 Serological Testing

Serological analyses were performed on samples collected at baseline, 4 weeks after the first and second ILIT injections, and at the summer visit in 2022 (2–4 months post‐ILIT dose). Modest increases in allergen‐specific immunoglobulin E (IgE) and immunoglobulin G4 (IgG4) were observed in both groups without clear treatment‐specific differences. Changes were consistent with seasonal variation rather than a distinct ILIT effect Figure 4C and Table S8.

## 4 Discussion

Intralymphatic immunotherapy with three low doses of a modified grass allergen formulated with MCT was well tolerated and resulted in reduced ARC‐associated symptoms and medication use during both the first and second pollen seasons following treatment, with significantly lower cSMS compared to placebo during the second pollen season. The European Association of Allergology and Clinical Immunology (EAACI) recommends cSMS as the standardised primary endpoint for randomised controlled ARC trials [52] A ≥ 20% improvement over placebo is commonly considered the threshold for clinical relevance in AIT trials, aligning with effort to harmonise outcome measures across studies. In the present trial, the observed 33%–50% reduction in cSMS versus placebo therefore clearly met the predefined criterion for clinical effect. Based on these findings, the study was extended with an open‐label phase in which participants originally assigned to placebo received active allergoid ILIT prior to the third pollen season. In this subsequent season, when all participants had received ILIT, no between‐group difference in cSMS was observed, further supporting the clinical efficacy of ILIT in the treatment of ARC.

However, the treatment effect differed between seasons, with statistical significance reached only in 2023. This variability is likely multifactorial. In particular, the 2022 pollen season was characterised by lower peak pollen exposure and a shorter high‐exposure period, and the predefined reporting window unfortunately began after the onset of the pollen season, potentially limiting sensitivity to detect treatment effects. In addition, in a proportion of participants, the final ILIT injection was administered shortly before the 2022 peak pollen period, which may have reduced the time available for full immune priming. Consistent with these observations, sensitivity analyses demonstrated a greater treatment effect under higher pollen exposure, supporting an exposure‐dependent efficacy of ILIT. This suggests that pollen load plays a significant role in the efficacy of ILIT.

Previous ILIT studies have primarily enrolled patients with seasonal allergic rhinitis caused by grass or tree pollen sensitisation and have typically used native allergen extracts adsorbed to aluminium‐based adjuvants [6, 8, 11, 13, 14, 15, 18]. Such products were typically marketed for monthly or co‐seasonal SCIT administration and repurposed for ILIT. In contrast, chemically modified allergens (allergoids) are routinely used for pre‐seasonal SCIT, allowing effective treatment with a limited number of injections over consecutive years [30, 31, 53]. The present study represents a phase IIa/b safety and proof‐of‐concept trial, and given that this is the first randomised controlled trial evaluating intralymphatic administration of a chemically modified grass pollen allergoid formulated with the biodegradable adjuvant MCT, the findings must be interpreted in light of the limited dose regimen and short treatment duration. However, the findings suggest that allergoids and MCT may also be well suited for ILIT and warrant further investigation, as their use could offer opportunities to improve both efficacy and safety.

To date, only one ILIT study has investigated MCT‐based formulations, administering cat, dog, or house dust mite allergens to patients with ARC [54]. In that trial, ILIT did not demonstrate a pronounced therapeutic effect on allergic rhinitis and was associated with moderate‐to‐severe systemic reactions and injection‐site pain. Several methodological differences may account for the discrepant findings. First, the study by Park et al. used RQLQ as the primary outcome measure, whereas EAACI recommends cSMS as the preferred primary endpoint in ARC trials; notably, in our study, treatment effects assessed by RQLQ (13%–22%) were also smaller than those observed using cSMS (33%–50%). Secondly, the study population was heterogeneous, including patients sensitised to cat, dog, or house dust mite allergens, and only 22 of 32 enrolled participants were included in the final efficacy analysis. Third, allergen dosing differed substantially; we administered three fixed doses of 133 SU (corresponding to 1/15 of a single SCIT maintenance dose), while Park et al. used a dose‐escalation scheme with markedly lower doses (0.4, 1.2, and 4 SU). Hence, we used a cumulative dose that was approx. 70‐fold higher. Consistent with these differences, injection‐site pain was the most frequently reported local AE in their study, followed by itchiness and wheal formation, while dyspnoea and wheezing were the most common systemic reactions. In contrast, we observed a higher frequency of local injection‐site reactions, likely related to the higher allergen doses used, whereas the incidence of systemic adverse events was comparable between studies. Pain was not assessed in our study.

Although ILIT has often been promoted on the premise that very low allergen doses are sufficient to induce clinical benefit, our data indicate that higher doses may be feasible without compromising safety. Future studies should, therefore, systematically explore optimal dosing strategies, including dose escalation and alternative dosing frequencies. Most ILIT trials to date have relied on three fixed‐dose injections. However, additional injections or modified schedules may further enhance treatment effects.

The relatively modest changes observed in allergen‐specific antibodies in the present study may support the notion that the dosing regimen remains suboptimal for inducing robust immunological responses. In conventional SCIT, effective treatment is typically associated with more pronounced increases in allergen‐specific IgG4, which are often predictive of long‐term clinical benefits. For example, a recent randomised phase III trial (*n* = 555) using the same MCT‐adjuvanted grass pollen allergoid, combined with monophosphoryl lipid A, demonstrated a strongly significant increase in grass pollen–specific IgG4 after six SCIT injections within 14 weeks, alongside a cSMS reduction of 0.27 points and a ≈20% improvement over placebo [55]. Notably, this significant immune response was achieved with a cumulative allergen dose of 27,600 SU, while we used 400 SU. Given the promising clinical signal observed with a lower allergen dose, our data underscore the potential for dose optimisation in future ILIT studies, which could improve both clinical outcomes and immunological markers. Moreover, given the apparent discrepancy between clinical improvement and little IgG4 induction, follow‐up studies should also investigate alternative mechanisms of protection, e.g., T‐cell modulation and local immune responses.

In conclusion, this study provides the first randomised clinical evidence supporting the safety and efficacy of ILIT using a grass pollen allergoid formulated with MCT. The observed clinical benefits compared to placebo are encouraging, but the study highlights the need for further optimisation of dosing regimens and longer‐term follow‐up. Ongoing analyses will address final study endpoints, including effects on asthma‐related outcomes and additional immunological parameters, to better define the role of allergoid‐based ILIT in the treatment of ARC.

## Author Contributions

L.Š., B.H.‐V., J.S., H.J.H. and P.J. conceptualised and designed the study. L.Š., H.J.H. and P.J. prepared documents for ethical approval and approval from Swiss medical agency. L.Š. and P.J. submitted the approval applications and registered the trial at ClinicalTrial.gov. L.Š. and S.F. performed all ILITs. L.Š., S.F., T.A., E.W., A.C., C.L. and P.S.‐G. acted as medical investigators during screening, treatments, data collection and follow up. L.Š., S.F., M.M., T.A., B.H.‐V., J.S., H.J.H. and P.J. analysed the data. T.M.K. provided financial support and acted as medical advisor on ILIT. L.Š. and P.J. wrote the first version of the paper. All authors contributed to the design and to critically revising the paper. All authors approved the last version before submission.

## Funding

The study was supported by grants from Stiftung für wissenschaftliche Forschung an der Universität Zürich (STEP‐2022‐002), the Innovation Fund Denmark (909000065B) and the University Hospital Zurich Innovationspool (INNOV‐00175). The study was further supported by operational funds to TMK from the University of Zurich and the University Hospital Zurich. The IMD and the SPT solutions used in this study were supplied free of charge by Allergy Therapeutics Ltd.

## Conflicts of Interest

H.J.H. is registered as the inventor of a patent on ILIT using 60 ng Phl p 5. T.M.K. has served as medical advisor for Allergy Therapeutics Ltd. Allergy Therapeutics provided the I.M.P. in this study. P.J. has received research funding from Allergy Therapeutics Ltd. for pre‐clinical research products from Allergy Therapeutics. P.S.‐G. has received speaker fees from A.L.K. Abello, Allergy Therapeutics Ltd. and Stallergenes. L.Š. and P.J. have received travel grants from A.T.L. Other researchers participating in this study have no study‐relevant conflicts of interest. No bias in the study is expected from conflicts of interest.

## Supporting information


**Figure S1:** Pollen seasons during study (A) Daily grass pollen counts (grains/m^3^) with season start/end (solid vertical lines) and peak period (hatched lines) for 2022–2024 in the greater Zurich area (B) Total daily‐average pollen counts summed over each pollen season (C) Duration of the peak season, defined as ≥ 4 consecutive days with > 50 grains/m^3^.
**Figure S2:** Sensitivity analysis of combined symptom–medication score (cSMS) according to pollen load. Upper panel: Daily mean cSMS trajectories over the 2022 (left) and 2023 (right) grass pollen seasons for allergen‐treated (blue) and placebo‐treated (red) groups. Solid black curves indicate daily grass pollen concentrations. Solid vertical black lines mark the full grass pollen season, and dotted vertical black lines indicate the peak pollen period. Lower panel: treatment effect estimates (cSMS; allergen minus placebo) with 95% confidence intervals for 2022 (dark blue) and 2023 (light blue), stratified by pollen load applied for analysis: total season versus days with pollen > 130 grains/m3.
**Figure S3:** Contribution of symptom and medication scores to treatment‐related differences in cSMS during the 2022 and 2023 grass pollen seasons. Daily cSMS values (A), symptom scores (B), and medication scores (C) are shown separately for the allergen‐treated (blue) and placebo‐treated (red) groups. Grey shaded areas indicate daily grass pollen concentrations. Solid vertical lines indicate the grass pollen season and dotted lines the peak pollen period. The panels on the right show the percentage reduction in seasonal subject‐level mean outcomes in the allergen group relative to placebo. Between‐group comparisons were performed using the Wilcoxon rank‐sum test.
**Figure S4:** Open Label Extension (A) cSMS and (B) RQLQ in the open label extension (15 May–15 Aug 2024) after ILIT with allergen in 2024 (green) or 2022 (blue). Smoothed cSMS and RQLQ with 95% CI plotted against log‐scaled daily pollen counts (grains/m3, grey area) over time for 2024. Pollen seasons are indicated by solid vertical lines, peak period by dotted lines. Treatment effect analyses for cSMS (Wilcoxon rank‐sum test) and RQLQ (Welch t‐test) based on subject‐level aggregates during peak pollen seasons showed no significant difference (3.9% favouring crossover with p = 0.59% and 3.9% favouring allergen with p = 0.19, respectively).
**Figure S5:** Subjective symptom improvement after pollen seasons. Subjective symptom scores were assessed after pollen seasons 2022 (V5) and 2023 (V7). Baseline symptom levels prior to treatment were set to 100%, and changes are shown relative to baseline (Δ); negative values indicate symptom improvement. Boxplots display the median and interquartile range, with whiskers representing 1.5 times the interquartile range and dots indicating individual participants. For between‐group comparisons Wilcoxon rank‐sum test was used. *p < 0.05.
**Figure S6:** Influence of pollen load on ILIT‐associated on lung function changes. (A) shows linear regressions between log‐transformed simple moving averages (SMA) of grass pollen counts and FeNO change from baseline in the placebo group during summer 2022 (left) and 2023 (right). (B) To estimate the effect of pollen load on ILIT efficacy, regression models were extended to include data from the ILIT group and additional covariates for summer 2022 (left) and summer 2023 (right). Dashed lines indicate the mean grass pollen counts during the respective follow‐up periods (summer 2022: 37.6 grains/m3; summer 2023: 124.9 grains/m3). ΔFeNO denotes the between‐group difference (allergen vs. placebo) in FeNO change from baseline at the mean pollen count of the follow‐up period. Grey shaded areas represent 95% confidence intervals.
**Figure S7:** Asthma subgroup analysis of ILIT efficacy. (A) Changes in FEV1% pred in non‐asthmatic (left) and asthmatic participants (right) and (B) changes in FeNO in non‐asthmatic (left) and asthmatic participants (right). FEV1% pred denotes FEV1 expressed as percentage of the predicted value. Boxplots show the 25th percentile, median, and 75th percentile; dots represent individual participants. Between‐group comparisons were performed using the Mann–Whitney U test. *p < 0.05.

**Table S1:** Adverse events (AEs) by injection number. Local injection‐site reactions (wheal/redness/swelling) and systemic AEs (generalised urticaria; chest pressure/dyspnoea) were recorded after each ILIT injection (V1–V3). AEs were assessed by physician 40 min post‐ILIT and self‐reported by participants at 40 min, end of day 0, and on days 1–3 post‐ILIT. Values represent the number of reported AEs per participant with completed questionnaires (percentage in brackets).
**Table S2:** Adverse event (AE) severity assessed by the physician. Local injection‐site reactions (Inj. site; wheal/redness/swelling) and systemic AEs, including generalised urticaria (Urticaria) and chest pressure or dyspnoea (Lung), were assessed after each ILIT injection (V1–3) and for the total of the three visits. AE severity (none, mild, or moderate) was evaluated by the physician 40 min post‐injection. Values are shown as *n*/*N* (%), where *N* is the number of participants with a completed symptom‐specific assessment at the respective visit. Percentages are based on available data; denominators may therefore differ slightly between symptoms. *p*‐values compare the proportion of participants with any adverse event (mild or moderate) between placebo ILIT and allergen ILIT.
**Table S3:** Combined symptom medication score (cSMS) analysis. Summary of cSMS outcomes for allergen and placebo ILIT groups, including the number of participants (*n*), total reporting days, median cSMS values with interquartile range (IQR), between‐group differences with Hodges–Lehmann (HL) confidence intervals (CI), relative reduction (%), and corresponding *p* values.
**Table S4:** Rhinitis Quality of Life Questionnaire (RQLQ) outcomes. Summary of RQLQ results for allergen and placebo ILIT groups, including the number of participants (*n*), total reporting weeks, median RQLQ scores, between‐group differences with confidence intervals (CI) and standard error (SE), relative reduction (%), and corresponding *p* values.
**Table S5:** Skin prick test (SPT) raw data and sensitivity analyses of the influence of ILIT on SPT responses. Wheal diameters to timothy grass and rye pollen allergens were measured at visits V5 and V7.
**Table S6:** Lung function and FeNO. Raw data and sensitivity analyses of the influence of ILIT on spirometry and FeNO. Lung function parameters (FEV1, FEV1% pred, FVC, FVC% pred, FEV1/FVC ratio) and exhaled nitric oxide (FeNO) were measured at visits V4–7, representing summer 2022 (V4), autumn/winter 2022 (V5), summer 2023 (V6), and autumn/winter 2023 (V7).
**Table S7:** Influence of pollen load on ILIT effects on FeNO. Multiple linear regression models were used to estimate the impact of grass pollen load on ILIT‐induced changes in FeNO. The outcome variable at V4 was FeNO change from baseline (V0) in ppb. For V6, a modified model was applied to avoid negative values, using log10‐transformed FeNO change from baseline plus an offset of +9 ppb. The intercept and the coefficient for “treatment group” are estimated at the mean grass pollen count of the respective follow‐up period (37.6 No/m3 for summer 2022, 124.9 No/m3 for summer 2023). SMA denotes simple moving averages of grass pollen counts. SE denotes standard error.
**Table S8:** Serum IgE and IgG4 antibodies. Antibody concentrations were measured by ImmunoCAP against toity grass allergen extract (g6), rye grass allergen extract (g12), Phl p 1 (g205), Phl p 5, (g215), and Phl 7/12 (g214). Results are given as median and range (min‐max) with sIgE reported as kUA/L and sIgG4 as μg/L. The median total IgE as measured at baseline (V1) was 94 kU/L (range: 19–574) for the group receiving placebo ILIT and 160 kU/L (range: 21–1365) for the group receiving allergen ILIT.

**Data S1:** Supporting information.

## Data Availability

The data that support the findings of this study are available from the corresponding author upon reasonable request.
