# Supplementary material for: Efficacy and Safety of Intralymphatic Immunotherapy With Grass Allergoid and Microcrystalline Tyrosine: A Double‐Blind Randomised Placebo‐Controlled Trial
Source: Allergy. 2026 Jul 29;81(11):4051–64. doi: 10.1111/all.70463 (PMC13753709; doi:10.1111/all.70463)
Supplement: Supplementary file 1 [file ALL-81-4051-s001.pdf]

Supplementary tables

Efficacy and safety of intralymphatic immunotherapy with grass allergoid and microcrystalline tyrosine: a double-blind randomised placebo-controlled trial

Lara Šošić et al.

Supplementary Table S1. Adverse events (AEs) by injection number. Local injection-site reactions (wheal/redness/swelling) and systemic AEs (generalised urticaria; chest pressure/dyspnoea) were recorded after each ILIT injection (V1–V3). AEs were assessed by physician 40 minutes post-ILIT and self-reported by participants at 40 minutes, end of day 0, and on days 1–3 post-ILIT. Values represent the number of reported AEs per participant with completed questionnaires (percentage in brackets).

ILIT	AE	Timepoint	Placebo ILIT		Allergen ILIT	
			Any AE	Moderate AE	Any AE	Moderate AE
ILIT no. 1 (V1)	Injection site	40 min (physician)	1/29 (3.5%)	0/29 (0.0%)	14/31 (45.2%)	1/31 (3.2%)
		40 min	1/29 (3.5%)	0/29 (0.0%)	19/31 (61.3%)	0/31 (0.0%)
		Day 0	1/25 (4.0%)	0/25 (0.0%)	11/31 (35.5%)	0/31 (0.0%)
		Day 1	1/29 (3.5%)	0/29 (0.0%)	7/31 (22.6%)	0/31 (0.0%)
		Day 2	1/28 (3.6%)	0/28 (0.0%)	5/29 (17.2%)	0/29 (0.0%)
		Day 3	0/28 (0.0%)	0/28 (0.0%)	1/31 (3.2%)	0/31 (0.0%)
	Urticaria	40 min (physician)	0/28 (0.0%)	0/28 (0.0%)	0/31 (0.0%)	0/31 (0.0%)
		40 min	0/29 (0.0%)	0/29 (0.0%)	1/31 (3.2%)	0/31 (0.0%)
		Day 0	0/25 (0.0%)	0/25 (0.0%)	2/31 (6.5%)	0/31 (0.0%)
		Day 1	0/29 (0.0%)	0/29 (0.0%)	3/31 (9.7%)	0/31 (0.0%)
		Day 2	1/28 (3.6%)	0/28 (0.0%)	1/29 (3.5%)	0/29 (0.0%)
		Day 3	1/28 (3.6%)	0/28 (0.0%)	1/31 (3.2%)	0/31 (0.0%)
	Lung	40 min (physician)	1/28 (3.6%)	0/28 (0.0%)	0/31 (0.0%)	0/31 (0.0%)
		40 min	1/29 (3.5%)	0/29 (0.0%)	0/31 (0.0%)	0/31 (0.0%)
		Day 0	0/25 (0.0%)	0/25 (0.0%)	1/31 (3.2%)	0/31 (0.0%)
		Day 1	1/29 (3.5%)	0/29 (0.0%)	0/31 (0.0%)	0/31 (0.0%)
		Day 2	1/28 (3.6%)	0/28 (0.0%)	0/29 (0.0%)	0/29 (0.0%)
		Day 3	1/28 (3.6%)	0/28 (0.0%)	2/31 (6.5%)	0/31 (0.0%)
ILIT no. 2 (V2)	Injection site	40 min (physician)	0/29 (0.0%)	0/29 (0.0%)	19/31 (61.3%)	1/31 (3.2%)
		40 min	2/29 (6.9%)	0/29 (0.0%)	18/31 (58.1%)	0/31 (0.0%)
		Day 0	2/29 (6.9%)	0/29 (0.0%)	18/31 (58.1%)	0/31 (0.0%)
		Day 1	2/28 (7.1%)	0/28 (0.0%)	12/31 (38.7%)	0/31 (0.0%)
		Day 2	1/28 (3.6%)	0/28 (0.0%)	6/31 (19.4%)	0/31 (0.0%)
		Day 3	0/26 (0.0%)	0/26 (0.0%)	4/31 (12.9%)	0/31 (0.0%)
	Urticaria	40 min (physician)	0/29 (0.0%)	0/29 (0.0%)	0/31 (0.0%)	0/31 (0.0%)
		40 min	0/29 (0.0%)	0/29 (0.0%)	1/31 (3.2%)	0/31 (0.0%)
		Day 0	0/27 (0.0%)	0/27 (0.0%)	0/30 (0.0%)	0/30 (0.0%)
		Day 1	1/28 (3.6%)	0/28 (0.0%)	0/31 (0.0%)	0/31 (0.0%)
		Day 2	1/28 (3.6%)	0/28 (0.0%)	0/31 (0.0%)	0/31 (0.0%)
		Day 3	1/26 (3.9%)	0/26 (0.0%)	0/31 (0.0%)	0/31 (0.0%)
	Lung	40 min (physician)	1/29 (3.5%)	0/29 (0.0%)	1/31 (3.2%)	0/31 (0.0%)
		40 min	1/29 (3.5%)	0/29 (0.0%)	1/31 (3.2%)	0/31 (0.0%)
		Day 0	1/28 (3.6%)	0/28 (0.0%)	1/31 (3.2%)	0/31 (0.0%)
		Day 1	1/28 (3.6%)	0/28 (0.0%)	0/31 (0.0%)	0/31 (0.0%)
		Day 2	0/28 (0.0%)	0/28 (0.0%)	0/30 (0.0%)	0/30 (0.0%)
		Day 3	0/26 (0.0%)	0/26 (0.0%)	0/31 (0.0%)	0/31 (0.0%)
ILIT no. 3 (V3)	Injection site	40 min (physician)	0/29 (0.0%)	0/29 (0.0%)	20/31 (64.5%)	1/31 (3.2%)
		40 min	0/29 (0.0%)	0/29 (0.0%)	19/31 (61.3%)	0/31 (0.0%)
		Day 0	2/28 (7.1%)	0/28 (0.0%)	17/31 (54.8%)	0/31 (0.0%)
		Day 1	0/26 (0.0%)	0/26 (0.0%)	14/31 (45.2%)	0/31 (0.0%)
		Day 2	0/27 (0.0%)	0/27 (0.0%)	6/29 (20.7%)	0/29 (0.0%)
		Day 3	0/27 (0.0%)	0/27 (0.0%)	2/30 (6.7%)	0/30 (0.0%)
	Urticaria	40 min (physician)	0/29 (0.0%)	0/29 (0.0%)	0/31 (0.0%)	0/31 (0.0%)
		40 min	0/29 (0.0%)	0/29 (0.0%)	0/31 (0.0%)	0/31 (0.0%)
		Day 0	0/28 (0.0%)	0/28 (0.0%)	0/29 (0.0%)	0/29 (0.0%)
		Day 1	0/26 (0.0%)	0/26 (0.0%)	0/31 (0.0%)	0/31 (0.0%)
		Day 2	0/27 (0.0%)	0/27 (0.0%)	1/29 (3.5%)	0/29 (0.0%)
		Day 3	0/27 (0.0%)	0/27 (0.0%)	0/30 (0.0%)	0/30 (0.0%)
	Lung	40 min (physician)	0/29 (0.0%)	0/29 (0.0%)	1/31 (3.2%)	0/31 (0.0%)
		40 min	1/29 (3.5%)	0/29 (0.0%)	1/31 (3.2%)	0/31 (0.0%)
		Day 0	0/28 (0.0%)	0/28 (0.0%)	1/29 (3.5%)	0/29 (0.0%)
		Day 1	1/25 (4.0%)	0/25 (0.0%)	0/31 (0.0%)	0/31 (0.0%)
		Day 2	0/27 (0.0%)	0/27 (0.0%)	0/29 (0.0%)	0/29 (0.0%)
		Day 3	0/27 (0.0%)	0/27 (0.0%)	0/30 (0.0%)	0/30 (0.0%)

Supplementary Table S2. Adverse event (AE) severity assessed by the physician. Local injection-site reactions (Inj. site; wheal/redness/swelling) and systemic AEs, including generalised urticaria (Urticaria) and chest pressure or dyspnoea (Lung), were assessed after each ILIT injection (V1–3) and for the total of the three visits. AE severity (none, mild, or moderate) was evaluated by the physician 40 minutes post-injection. Values are shown as n/N (%), where N is the number of participants with a completed symptom-specific assessment at the respective visit. Percentages are based on available data; denominators may therefore differ slightly between symptoms. P-values compare the proportion of participants with any adverse event (mild or moderate) between placebo ILIT and allergen ILIT.

ILIT	AE	Placebo ILIT			Allergen ILIT			P value
		Mild	Moderate	None	Mild	Moderate	None	
V1	Inj. site	1/29 (3.5%)	0/29 (0.0%)	28/29 (96.6%)	13/31 (41.9%)	1/31 (3.2%)	17/31 (54.8%)	<0.001
	Urticaria	0/280 (0.0%)	0/280 (0.0%)	28/28 (100%)	0/310 (0.0%)	0/31 (0.0%)	31/3131 (100%)	1.0
	Lung	1/28 (3.6%)	0/28 (0.0%)	27/28 (96.4%)	0/30 (0.0%)	0/31 (0.0%)	31/31 (100%)	0.48
V2	Inj. site	0/29 (0.0%)	0/29 (0.0%)	29/29 (100%)	18/31 (58.1%)	1/31 (3.2%)	12/31 (38.7%)	<0.001
	Urticaria	0/29 (0.0%)	0/29 (0.0%)	29/29 (100%)	0/31 (0.0%)	0/31 (0.0%)	31/31 (100%)	1.0
	Lung	1/29 (3.4%)	0/29 (0.0%)	28/29 (96.6%)	1/31 (3.2%)	0/31 (0.0%)	30/31 (96.8%)	1.0
V3	Inj. site	0/29 (0.0%)	0/29 (0.0%)	29/29 (100%)	19/31 (61.3%)	1/31 (3.2%)	11/31 (35.5%)	<0.001
	Urticaria	0/29 (0.0%)	0/29 (0.0%)	29/29 (100%)	0/31 (0.0%)	0/31 (0.0%)	31/31 (100%)	1.0
	Lung	0/29 (0.0%)	0/29 (0.0%)	29/29 (100%)	1/31 (3.2%)	0/31 (0.0%)	30/31 (96.8%)	1.0
Total	Inj. site	1/29 (3.4%)	0/29 (0.0%)	28/29 (96.6%)	13/31 (41.9%)	1/31 (3.2%)	17/31 (54.8%)	<0.001
	Urticaria	0/28 (0.0%)	0/28 (0.0%)	28/28 (100%)	0/31 (0.0%)	0/31 (0.0%)	31/31 (100%)	1.0
	Lung	1/28 (3.6%)	0/28 (0.0%)	27/28 (96.4%)	0/31 (2.2%)	0/31 (0.0%)	31/31 (100%)	1.0

Supplementary Table S3. Combined symptom medication score (cSMS) analysis. Summary of cSMS outcomes for allergen and placebo ILIT groups, including the number of participants (n), total reporting days, median cSMS values with interquartile range (IQR), between-group differences with Hodges–Lehmann (HL) confidence intervals (CI), relative reduction (%), and corresponding p values.

Measured statistical parameter	Year		
	**2022**	**2023**	**2024**
Allergen, n=	31	31	19
Allergen reporting days	2634	2955	1805
Allergen median	0.33	0.33	0.42
Allergen IQR	0.17–0.83	0.17–0.83	0.17–1.33
Placebo, n=	29	28	26
Placebo reporting days	2400	2570	2187
Placebo median	0.5	0.67	0.38
Placebo IQR	0.33–1.00	0.33–1.00	0.00–1.00
Difference	-0.17	-0.25	0.08
HL CI	-0.33 – 0.17	-0.50 – 0.00	-0.17 – 0.33
Reduction (%)	33.33	50.00	-11.11
P-value	0.26	0.04	0.59

Supplementary Table S4. Rhinitis Quality of Life Questionnaire (RQLQ) outcomes. Summary of RQLQ results for allergen and placebo ILIT groups, including the number of participants (n), total reporting weeks, median RQLQ scores, between-group differences with confidence intervals (CI) and standard error (SE), relative reduction (%), and corresponding P values.

**Measured statistical parameter**	**Year**		
	**2022**	**2023**	**2024**
Allergen, n=	31	31	19
Allergen reporting weeks	349	416	240
Allergen mean	0.86	0.91	0.72
Placebo, n=	29	28	26
Placebo reporting weeks	316	343	295
Placebo median	1.09	1.05	0.96
Difference	-0.23	-0.14	-0.24
Confidence interval (CI)	-0.54–0.07	-0.47–0.20	-0.62–0.13
Std. error	0.15	0.17	0.18
Reduction (%)	21.54	12.89	25.21
P-value	0.12	0.43	0.19

Supplementary Table S5. Skin prick test (SPT) raw data and sensitivity analyses of the influence of ILIT on SPT responses. Wheal diameters to timothy grass and rye pollen allergens were measured at visits V5 and V7.

Outcome, specificity, visit no.	Placebo Change ± SD [a]	Allergen Change ± SD [a]	Difference (95% CI) [b]	Adjusted diff. (95% CI) [c]
**SPT wheal ∅ (mm)**				
Timothy (V5)	-1.9 ± 2.9	-1.9 ± 4.0	-0.0 (-1.8–1.8)	-0.2 (-1.8–1.5)
Timothy (V7)	-1.9 ± 4.5	-1.0 ± 3.7	+0.9 (-1.3–3.1)	+0.7 (-1.5–2.9)
Rye V5	-0.8 ± 4.2	-2.1 ± 4.0	-1.3 (-3.4–0.8)	-1.2 (-2.8–0.5)
Rye V7	-2.9 ± 4.3	-1.9 ± 4.3	+1.0 (-1.3–3.3)	+1.1 (-1.3–3.3)
**C_{ha} – (SU/ml)**				
Timothy (V5)	2.32×10^{11} ± 1.16×10^{12}	1.48×10^{11} ± 5.32×10^{10}	-8.12×10^{10} (-5.62×10^{11} - 3.95×10^{11}	-
Timothy (V7)	3.51×10^{10} ± 4.46×10^{10}	8.29×10^{10} ±1.79×10^{3}	4.78×10^{10} (-1.35×10^{11}–2.30×10^{11})	-
Rye (V5)	4.14×10^{9} ± 5.93×10^{10}	2.06×10^{10} ± 1.05×10^{11}	1.65×10^{10} (-2.07×10^{10}–4.14×10^{10})	-
Rye (V7)	4.62×10^{9} ± 6.28×10^{10}	3.87×10^{2} ± 1.62×10^{3}	-4.62×10^{9} (-3.0×10^{10}–2.08×10^{10})	-

a) Mean relative change from baseline (V0, winter 2022). Values are presented as mean ± SD.

b) Difference in mean change from baseline between placebo and ILIT. Due to rounding, this value may not exactly match the table's mean change values. Change-from-baseline comparisons between groups were performed using Mann-Whitney U-tests. 95% CIs are provided for reference only and not for hypothesis testing.

c) Difference in adjusted mean change from baseline between placebo and ILIT, estimated using ANCOVA models adjusting for covariate.

Supplementary Table S6. Lung function and FeNO. Raw data and sensitivity analyses of the influence of ILIT on spirometry and FeNO. Lung function parameters (FEV1, FEV1%pred, FVC, FVC%pred, FEV1/FVC ratio) and exhaled nitric oxide (FeNO) were measured at visits V4–7, representing summer 2022 (V4), autumn/winter 2022 (V5), summer 2023 (V6), and autumn/winter 2023 (V7).

Outcome, specificity, visit no.	Placebo Change ± SD [a]	Allergen Change ± SD [a]	Difference [b] (95% CI)	Adjusted diff. [c] (95% CI)	p
**FEV1 (L)**					
V4	-0.13 ± 0.20	-0.07 ± 0.27	0.06 (-0.07–0.18)	0.03 (-0.10–0.16)	
V5	-0.13 ± 0.27	-0.02 ± 0.26	0.12 (-0.02–0.25)	0.12 (-0.02–0.27)	0.09
V6	-0.11 ± 0.22	-0.03 ± 0.31	0.08 (-0.06–0.22)	0.07 (-0.08–0.22)	
V7	-0.07 ± 0.22	0.02 ± 0.24	0.10 (-0.03–0.22)	0.11 (-0.02–0.24)	0.09
**FEV1pred (%)**					
V4	-2.7 ± 5.6	-0.2 ± 6.0	2.5 (-0.5–5.5)	2.1 (-1.0–5.1)	0.07
V5	-3.1 ± 7.3	0.6 ± 6.5	3.6 (0.1–6.0)	3.9 (0.2–7.6)	0.04
V6	-2.3 ± 5.9	0.7 ± 7.8	3.0 (-0.6–6.6)	2.2 (-1.5–6.0)	0.11
V7	-1.0 ± 5.9	2.4 ± 6.3	3.5 (0.1–6.8)	3.9 (0.3–7.4)	0.03
**FVC (L)**					
V4	-0.09 ± 0.26	-0.02 ± 0.34	0.07 (-0.08–0.23)	0.07 (-0.10–0.23)	
V5	-0.21 ± 0.32	-0.10 ± 0.25	0.11 (-0.04–0.26)	0.15 (-0.00–0.29)	0.05
V6	-0.22 ± 0.26	-0.17 ± 0.28	0.05 (-0.09–0.19)	0.08 (-0.05–0.22)	
V7	-0.02 ± 0.27	0.01 ± 0.35	0.03 (-0.14–0.20)	0.04 (-0.14–0.22)	
**FVCpred (%)**					
V4	-1.3 ± 5.3	-0.4 ± 6.9	1.0 (-2.2–4.1)	1.0 (-2.3–4.3)	
V5	-3.8 ± 6.2	-2.0 ± 5.3	1.8 (-1.2–4.8)	2.8 (-0.1–5.6)	0.06
V6	-3.7 ± 5.6	-3.0 ± 5.3	0.7 (-2.2–3.7)	1.4 (-1.4–4.2)	
V7	0.1 ± 5.2	0.8 ± 6.9	0.7 (-2.6–4.0)	1.1 (-2.4–4.7)	
**FEV1/FVC**					
V4	-0.01 ± 0.04	-0.01 ± 0.07	+0.00 (-0.02–0.03)	-0.00 (-0.03–0.02)	
V5	+0.01 ± 0.06	+0.01 ± 0.06	-0.00 (-0.03–0.03)	-0.01 (-0.03–0.03)	
V6	+0.02 ± 0.05	+0.02 ± 0.06	+0.00 (-0.03–0.03)	-0.00 (-0.03–0.02)	
V7	-0.01 ± 0.04	+0.00 ± 0.04	+0.02 (-0.00–0.04)	+0.02 (-0.00–0.04)	
**FeNO (ppb)**					
V4	+24.9 ± 20.8	+14.3 ± 21.1	-10.6 (-21.0–0.2)	-6.7 (-19.5–6.0)	0.07
V5	+6.8 ± 7.9	+6.8 ± 12.3	+0.1 (-5.3–5.4)	-3.9 (-9.7–1.8)	
V6	+30.6 ± 38.3	+24.4 ± 35.9	-6.2 (-25.8–13.5)	+0.7 (-22.9–24.2)	
V7	+5.8 ± 10.6	+4.7 ± 14.7	-1.2 (-8.0–5.7)	-1.9 (-10.1–6.3)	

a) Mean relative change from baseline (V0, winter 2022). Values are presented as mean ± SD.

b) Difference in mean change from baseline between placebo and ILIT. Due to rounding, this value may not exactly match the table's mean change values. Change-from-baseline comparisons between groups were performed using Mann-Whitney U-tests. 95% CIs are provided for reference only and not for hypothesis testing.

c) Difference in adjusted mean change from baseline between placebo and ILIT, estimated using ANCOVA models adjusting for covariate.

Supplementary Table S7. Influence of pollen load on ILIT effects on FeNO. Multiple linear regression models were used to estimate the impact of grass pollen load on ILIT-induced changes in FeNO. The outcome variable at V4 was FeNO change from baseline (V0) in ppb. For V6, a modified model was applied to avoid negative values, using log10-transformed FeNO change from baseline plus an offset of +9 ppb. The intercept and the coefficient for "treatment group" are estimated at the mean grass pollen count of the respective follow-up period (37.6 No/m³ for summer 2022, 124.9 No/m³ for summer 2023). SMA denotes simple moving averages of grass pollen counts. SE denotes standard error.

**Parameters**	**Summer 2022 (V4)**				**Summer 2023 (V6)**			
	Estimate	SE	t	p	Estimate	SE	t	p
Intercept	23.6	6.0	3.9	<0.001	1.63	0.15	11.04	<0.001
Log_{10}(SMA of grass pollen count (No/m³))	28.2	10.8	2.6	0.012	0.69	0.20	3.44	0.001
Treatment group: ILIT	-19.8	5.9	-3.4	0.001	-0.20	0.13	-1.56	0.126
FeNO baseline value (ppb)	0.4	0.2	2.0	0.048	-0.00	0.00	-0.57	0.574
Smoker at baseline: yes	-7.4	6.5	-1.1	0.260	0.01	0.17	0.05	0.959
Interaction between log_{10}(SMA of grass pollen count) and ILIT	-47.4	14.4	-3.3	0.002	-0.51	0.40	-1.29	0.204
Residual SE	17.59				0.4393			
Degrees of freedom	53				51			
Adjusted R^2:	0.198				0.126			
F-statistic	3.86 on 5 (p =0.005)				2.61 on 5 (p=0.035)			

Supplementary Table S8. Serum IgE and IgG4 antibodies. Antibody concentrations were measured by ImmunoCAP against toity grass allergen extract (g6), rye grass allergen extract (g12), Phl p 1 (g205), Phl p 5, (g215), and Phl 7/12 (g214). Results are given as median and range (min-max) with sIgE reported as kUA/L and sIgG4 as µg/L. The median total IgE as measured at baseline (V1) was 94 kU/L (range: 19–574) for the group receiving placebo ILIT and 160 kU/L (range: 21–1365) for the group receiving allergen ILIT.

CAP	Visit	Allergen		Placebo	
		sIgE	sIgG4	sIgE	sIgG4
Timothy grass	V1	15.7 (0.7–130.8)	0.3 (0.1–2.8)	15.4 (1.1–79.9)	0.5 (0.1–2)
	V2	20.6 (0.5–145.8)	0.2 (0.1–3)	16 (1–82.6)	0.4 (0.1–2.3)
	V3	16.6 (0.6–149.6)	0.2 (0.1–3.1)	13.6 (2.2–84)	0.4 (0.1–2.1)
	V4	39.4 (1–156.7)	0.3 (0.1–2.5)	24.5 (1.7–120.2)	0.4 (0.1–2.6)
	V5	36.4 (0.7–695.6)	0.3 (0.1–2.3)	17 (2.2–143.5)	0.3 (0.1–1.9)
	V6	19.2 (0.8–406.3)	0.2 (0.1–2)	15 (1.8–116.05)	0.3 (0–1.7)
	V7	23.2 (0.7–345.7)	0.3 (0–1.9)	23.6 (2.6–288.4)	0.4 (0.1–2.3)
Rye grass	V1	10.6 (0.5–69.7)	0.1 (0–2.1)	11.8 (0.6–58.5)	0.2 (0–1.4)
	V2	13.1 (0.4–85.6)	0.1 (0–2.3)	11.7 (0.5–58.4)	0.2 (0–1.7)
	V3	10.8 (0.4–122.3)	0.1 (0–2.4)	10 (1.1–62.1)	0.2 (0–1.7)
	V4	24.6 (0.7–114.9)	0.2 (0–2.1)	15.5 (1.1–81.8)	0.2 (0–2)
	V5	26.7 (0.8–184.3)	0.2 (0.1–2.3)	14 (1.9–91.8)	0.2 (0–2.5)
	V6	18.2 (0.8–232)	0.2 (0.1–2.2)	11.4 (1.5–91.2)	0.2 (0–2.2)
	V7	16.8 (0.8–242.5)	0.3 (0.1–2)	17.8 (2.1–318.7)	0.29 (0–2.6)
Ph l p 1	V1	10.4 (0.4–50.4)	0 (0–4.8)	8.5 (0.30–36)	0 (0–0.4)
	V2	8.9 (0.3–52.9)	0 (0–7.8)	8.6 (0.2–50.9)	0 (0–0.5)
	V3	7.7 (0.3–53.4)	0 (0–5.4)	7.4 (0.3–55.4)	0 (0–0.5)
	V4	17.8 (0.6–108.5)	0 (0–5.1)	12.1 (1.1–69.6)	0 (0–0.2)
	V5	15.6 (0.7–213.1)	0.1 (0–6.8)	11.8 (0.8–154.7)	0.1 (0–0.6)
	V6	9.8 (0.5–178.1)	0.1 (0–5.7)	10.6 (0.6–81)	0.1 (0–0.5)
	V7	11.1 (0.7–335.8)	0.1 (0–5.8)	16.6 (0.8–191.5)	0.1 (0–0.8)
Phl p 5	V1	6.7 (0–100.1)	0 (0–4)	6.6 (0–49.5)	0 (0–0.6)
	V2	7.8 (0–121.9)	0 (0–3.8)	6.6 (0–50.2)	0 (0–0.7)
	V3	8.6 (0–148.3)	0 (0–4.6)	5.2 (0–55)	0 (0–0.6)
	V4	19.3 (0–139.9)	0 (0–4.9)	12.2 (0–67.4)	0 (0–1.6)
	V5	20 (0–334)	0.1 (0–4.2)	8.3 (0–76)	0 (0–0.8)
	V6	12 (0–273.9)	0 (0–4.4)	5.8 (0–54.7)	0 (0–0.8)
	V7	10.4 (0–341.5)	0 (0–4.2)	12.3 (0–96.6)	0 (0–1.1)
Phl p 7/12	V1	0 (0–3.1)	0 (0–2.7)	0 (0–14.1)	0 (0–1.5)
	V2	0 (0–3.4)	0 (0–2.8)	0 (0–18.8)	0 (0–1.7)
	V3	0 (0–6.1)	0 (0–3.3)	0.1 (0–19.7)	0 (0–1.5)
	V4	0.1 (0–21.1)	0 (0–2.6)	0 (0–31.5)	0 (0–1.9)
