# Supplementary material for: Efficacy and Safety of Intralymphatic Immunotherapy With Grass Allergoid and Microcrystalline Tyrosine: A Double‐Blind Randomised Placebo‐Controlled Trial
Source: Allergy. 2026 Jul 29;81(11):4051–64. doi: 10.1111/all.70463 (PMC13753709; doi:10.1111/all.70463)
Supplement: Supplementary file 2 [file ALL-81-4051-s002.pdf]

Supplementary text

Efficacy and safety of intralymphatic immunotherapy with grass allergoid and microcrystalline tyrosine: a double-blind randomised placebo-controlled trial

Lara Šošić et al.

The investigational medicinal product Polvac® Grasses + Rye

The allergen extract used in this study is POLVAC® Grasses + Rye. The allergoid product is available in Switzerland. The product contains 13 grass species that represent a broad spectrum of clinically relevant grass pollen allergens in Switzerland and other European countries. The following 13 allergens are contained in the drug:

- Foxtail (*Alopecurus pratensis*)
- Crested Dogstail (*Cynosurus cristatus*)
- Cocksfoot (*Dactylis glomerata*)
- Rye grass (*Lolium perenne*)
- Meadow grass (*Poa pratensis*)
- Vernal (*Anthoxanthum odoratum*)
- Fescue (*Festuca pratensis*)
- Bent (*Agrostis tenuis*)
- Timothy (*Phleum pratense*)
- Brome (*Bromus spp.*)
- Oat grass (Ar*rhenatherum elatius*)
- Yorkshire Fog (*Holcus lanatus*)
- Cultivated Rye (*Secale cereale*).

The relative amount of each allergen is described by Alan Bullimore et al. in "*Cross-reactivity in Grasses: Biochemical Attributes Define Exemplar Relevance*", World Allergy Organ J. 2012 Oct;5(10):111-9. (doi: 10.1097/WOX.0b013e31826a10cf).

Allergic rhinitis symptom medication

Study participants were provided with standard medication for the treatment of allergic rhinitis symptoms and received detailed instructions on their appropriate use. Medication was available ad libitum during the periods of symptom and medication recording for the primary endpoint (cSMS). The following medications were supplied:

- Desloratadine 5 mg tablets (Aerius®, Merck & Co.)
- Mometasone furoate nasal spray (Nasonex®, Merck & Co.)
- Olopatadine eye drops (Opatanol®, Novartis)

Emergency medication

In addition to standard symptom-relieving medication, participants were provided with an emergency medication set consisting of:

- Two levocetirizine 5 mg tablets (Xyzal®, UCB)
- Two prednisolone 50 mg tablets (Spiricort®, Spirig HealthCare)

Participants were instructed on the appropriate use of emergency medication.

Combined symptom medication score (cSMS) recording

Participants were instructed on both the use of symptom-relieving medication and the recording of symptoms and medication intake via daily electronic questionnaires as described by EAACI in O. Pfaar et al., "*Recommendations for the standardization of clinical outcomes used in allergen immunotherapy trials for allergic rhinoconjunctivitis: an EAACI Position Paper*". Allergy. 2014 Jul;69(7):854-67 (doi: 10.1111/all.12383). The questionnaires were delivered via mobile phone (text message link) on a daily basis during the pollen seasons, from May 15 (2022) or April 15 (2023 and 2024) until the end of the observation period (August 15).

A screenshot of the original German daily symptom questionnaire is provided further below for reference. For the calculation of symptom score (0-1-2-3) the following and established system was applied:

- Score 0: no symptoms
- Score 1: mild symptoms (sign/symptom clearly present, but minimal awareness; easily tolerated)
- Score 2: moderate symptoms (definite awareness of sign/symptom that is bothersome but tolerable)
- Score 3: severe symptoms (sign/symptom that is hard to tolerate; causes interference with activities of daily living and/or sleeping)

In addition to symptoms, the participants also recorded daily medication use by answering "yes" or "no" to the following items:

- Use of oral antihistamines (Aerius® or Xyzal®)
- Use of antihistamine eye drops (Opatanol®)
- Use of intranasal corticosteroids (Nasonex®)
- Use of systemic corticosteroids (Spiricort®)
- Use of any other treatment for allergic symptoms

If participants answered "yes" to the last item, they were prompted to provide a free-text description of the additional treatment used. A screenshot of the original German daily questionnaire is provided further below for reference. For the calculation of the medication score (0-1-2-3), the following and established system was applied:

- Score 0: no medication used
- Score 1: oral and/or ocular antihistamines
- Score 2: intranasal corticosteroids with/without antihistamines
- Score 3: oral corticosteroids with/without intranasal corticosteroids, with/without antihistamines

Print scree of the original German daily questionnaire for symptoms

**Geben Sie an, wie Sie Ihre nasalen Symptome wahrnehmen.**				
	0 Keine Symptome	1	2	3 Sehr schwere Symptome
Juckreiz in der Nase	◯	◯	◯	◯
Niesen	◯	◯	◯	◯
Laufende Nase	◯	◯	◯	◯
Verstopfte Nase	◯	◯	◯	◯

**Geben Sie an, wie Sie Ihre Augensymptome wahrnehmen.**				
	0 Keine Symptome	1	2	3 Sehr schwere Symptome
Juckreiz in den Augen	◯	◯	◯	◯
Tränende Augen	◯	◯	◯	◯

**Bitte geben Sie an, inwieweit Sie den folgenden Aussagen zustimmen:**				
	0Trifft überhaupt nicht zu	1Nicht einverstanden	2Einverstanden	3Stimme voll und ganz zu
Ich fühle mich aufgrund meiner Symptome müde	◯	◯	◯	◯
Ich habe aufgrund meiner Symptome Konzentrationsschwierigkeiten	◯	◯	◯	◯

Print screen of the original German daily questionnaire for medication use:

**Geben Sie an, welche Medikamente Sie verwenden**		
	Nej	Ja
Antihistamin-Tablette (z.B. Aerius oder Xyzal)	◯	◯
Antihistamin-Augentropfen (z.B. Opatanol)	◯	◯
Kortisonhaltiger Nasenspray (z.B. Nasonex)	◯	◯
Kortison-Tablette (z.B. Spiricort)	◯	◯
Haben Sie etwas anderes getan, um Ihre Symptome zu lindern? (z. B. andere Medikamente, andere Behandlung usw.)	◯ Ja<br>◯ Nein	
Beschreiben Sie, was Sie sonst noch getan haben, um Ihre Symptome zu lindern	______________	
