# Supplementary material for: Efficacy and Safety of Intralymphatic Immunotherapy With Grass Allergoid and Microcrystalline Tyrosine: A Double‐Blind Randomised Placebo‐Controlled Trial
Source: Allergy. 2026 Jul 29;81(11):4051–64. doi: 10.1111/all.70463 (PMC13753709; doi:10.1111/all.70463)
Supplement: Supplementary file 3 [file ALL-81-4051-s003.pdf]

Supplementary figures

Efficacy and safety of intralymphatic immunotherapy with grass allergoid and microcrystalline tyrosine: a double-blind randomised placebo-controlled trial

Lara Šošić et al

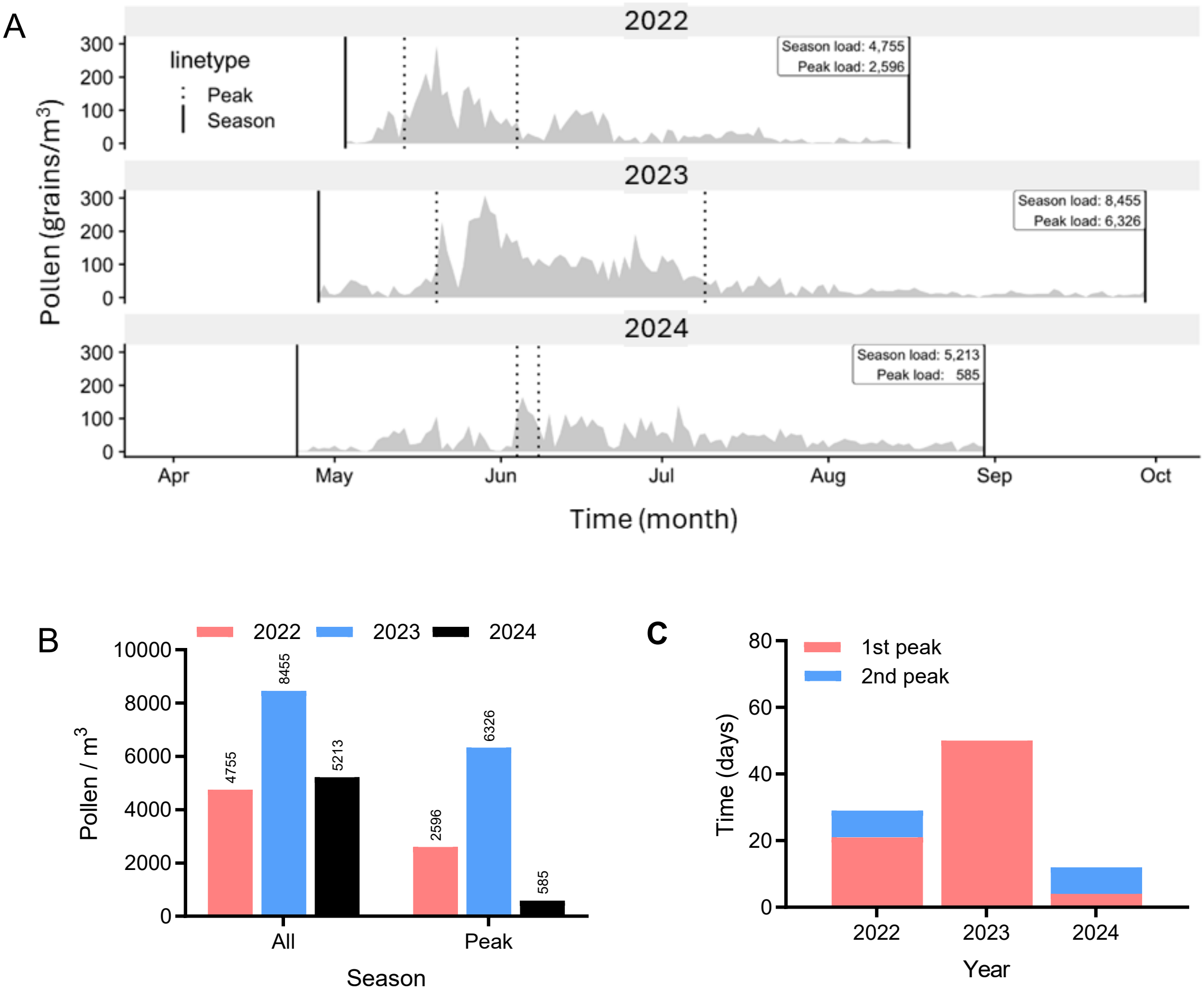

Supplementary Figure S1. Pollen seasons during study. **(A)** Daily grass pollen counts (grains/m³) with season start/end (solid vertical lines) and peak period (hatched lines) for 2022–2024 in the greater Zurich area. **(B)** Total daily-average pollen counts summed over each pollen season. **(C)** Duration of the peak season, defined as ≥4 consecutive days with >50 grains/m³.

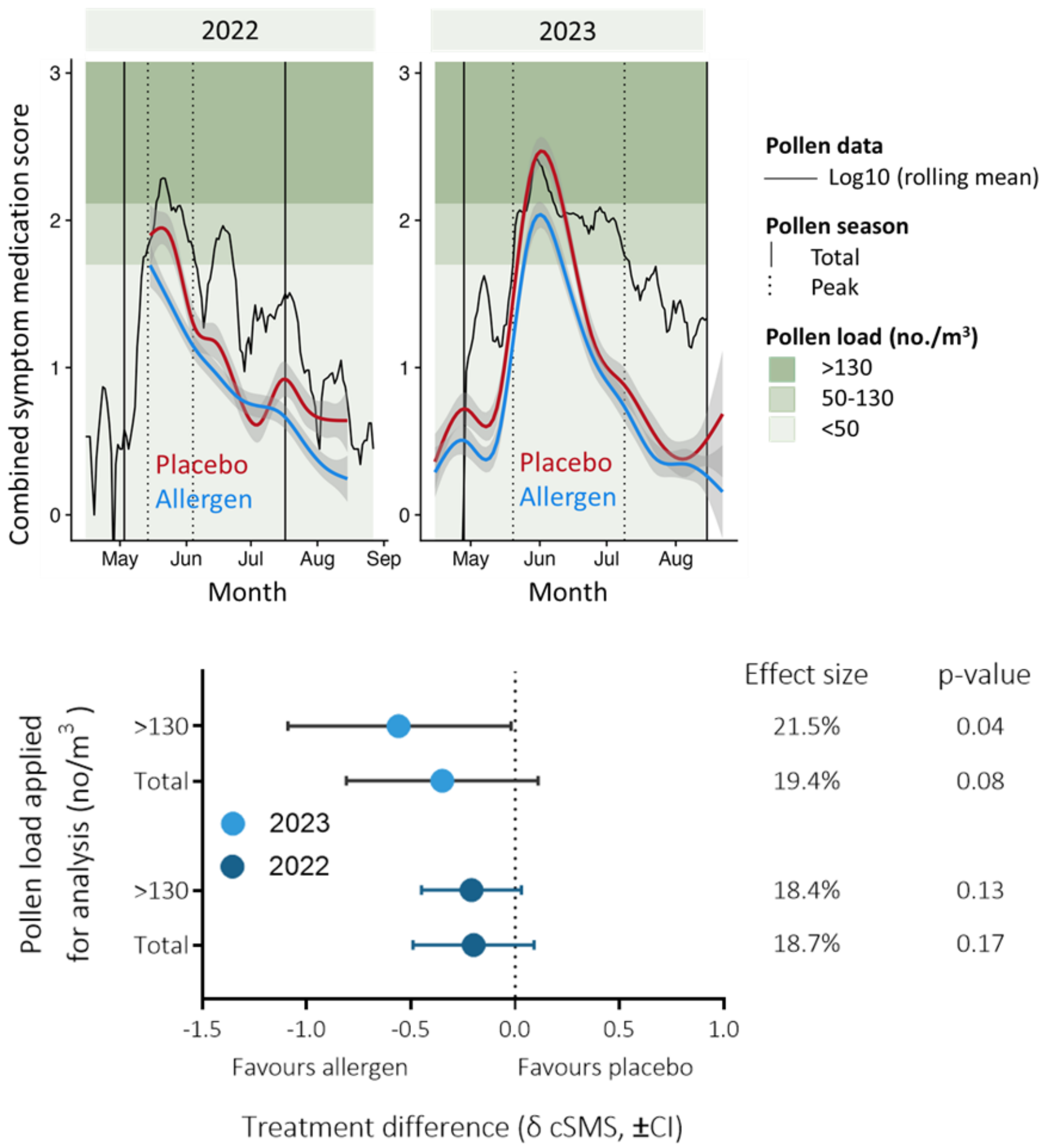

Supplementary Figure S2. Sensitivity analysis of combined symptom–medication score (cSMS) according to pollen load. *Upper panel:* Daily mean cSMS trajectories over the 2022 (left) and 2023 (right) grass pollen seasons for allergen-treated (blue) and placebo-treated (red) groups. Solid black curves indicate daily grass pollen concentrations. Solid vertical black lines mark the full grass pollen season, and dotted vertical black lines indicate the peak pollen period. *Lower panel:* treatment effect estimates (δcSMS; allergen minus placebo) with 95% confidence intervals for 2022 (dark blue) and 2023 (light blue), stratified by pollen load applied for analysis: total season versus days with pollen >130 grains/m³.

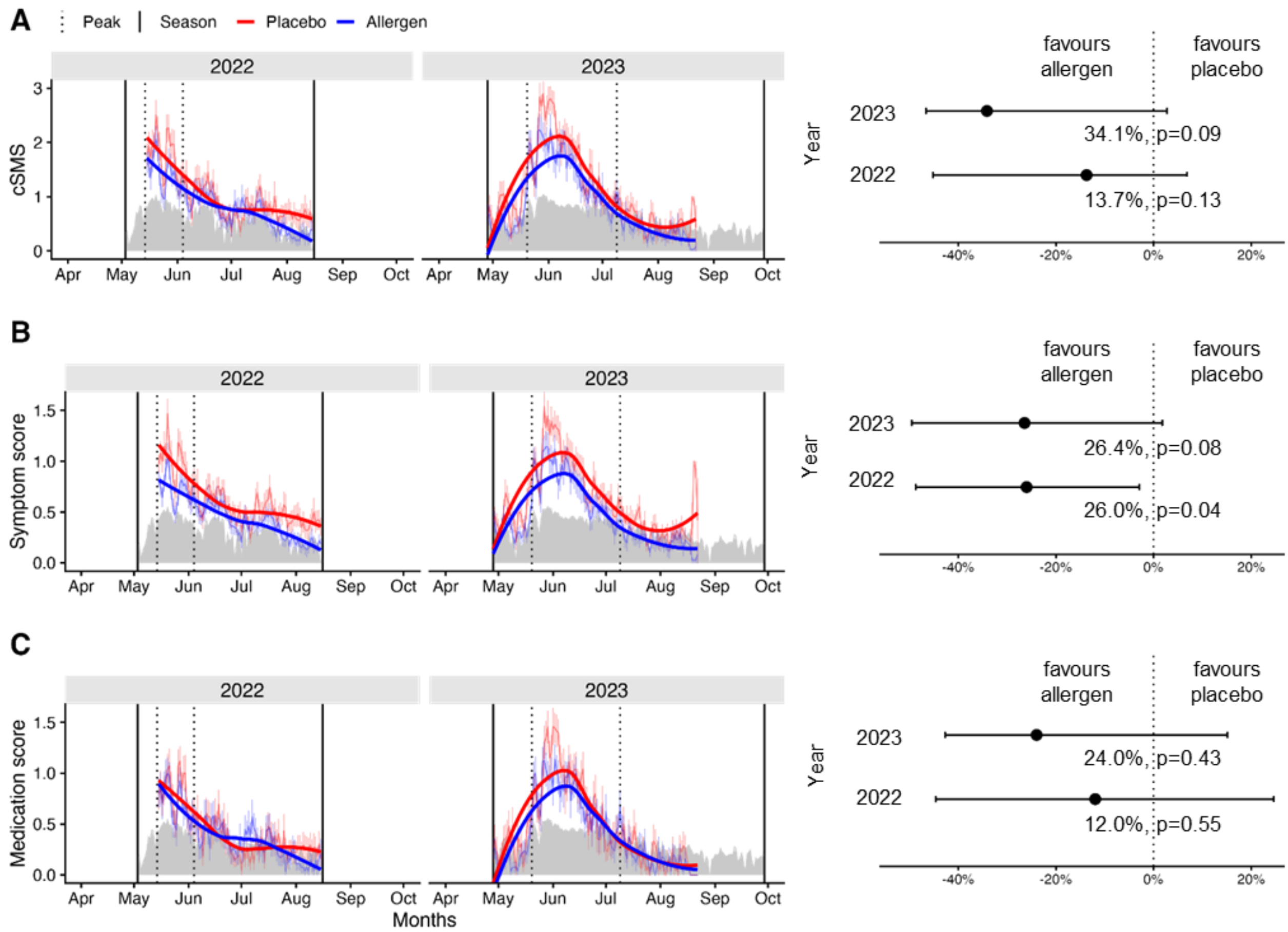

Supplementary Figure S3. Contribution of symptom and medication scores to treatment-related differences in cSMS during the 2022 and 2023 grass pollen seasons. Daily cSMS values (**A**), symptom scores (**B**), and medication scores (**C**) are shown separately for the allergen-treated (blue) and placebo-treated (red) groups. Grey shaded areas indicate daily grass pollen concentrations. Solid vertical lines indicate the grass pollen season and dotted lines the peak pollen period. The panels on the right show the percentage reduction in seasonal subject-level mean outcomes in the allergen group relative to placebo. Between-group comparisons were performed using the Wilcoxon rank-sum test.

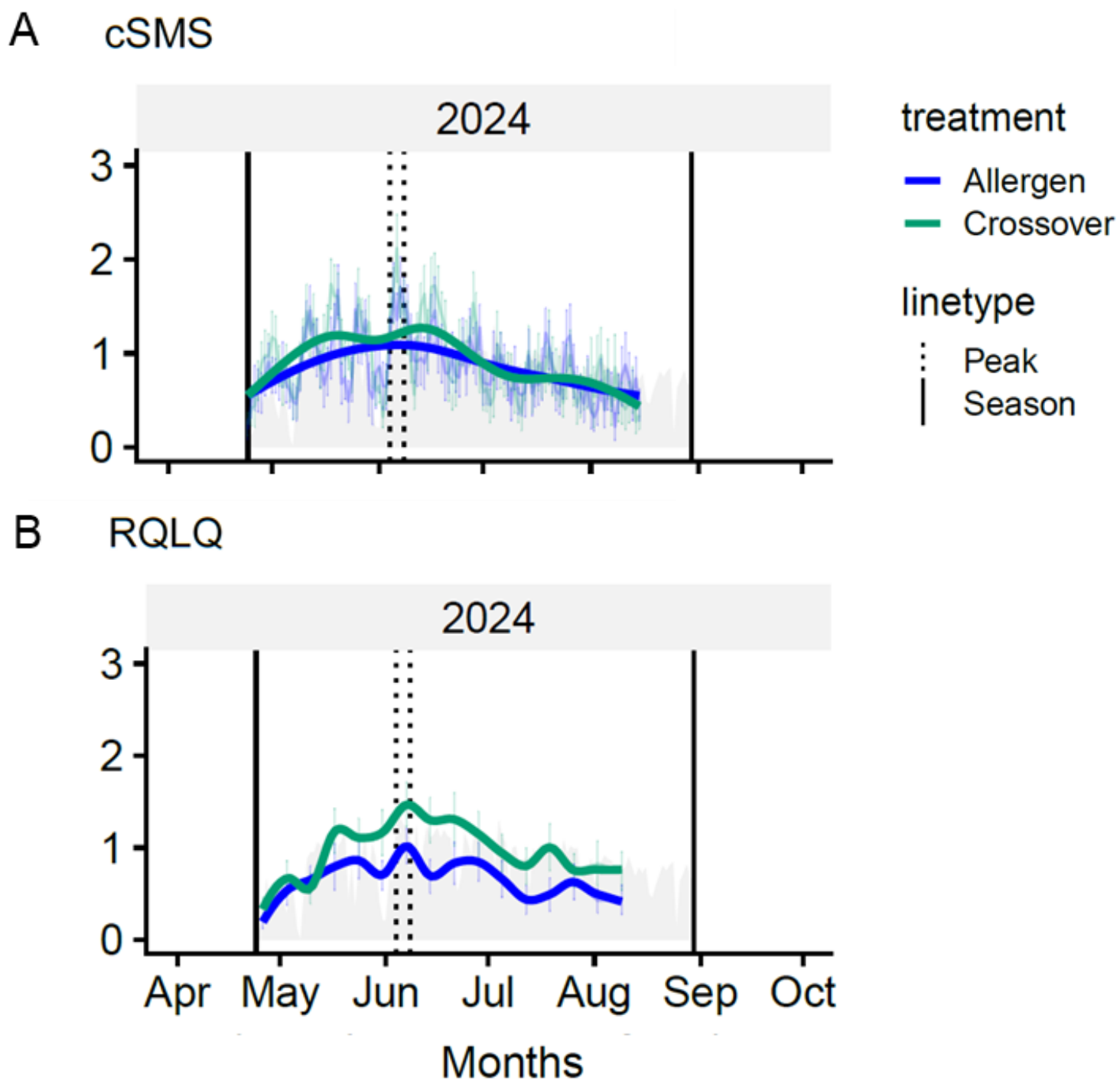

Supplementary Figure S4. Open Label Extension. (A) cSMS and **(B)** RQLQ in the open label extension (15 May–15 Aug 2024) after ILIT with allergen in 2024 (green) or 2022 (blue). Smoothed cSMS and RQLQ with 95% CI plotted against log-scaled daily pollen counts (grains/m³, grey area) over time for 2024. Pollen seasons are indicated by solid vertical lines, peak period by dotted lines. Treatment effect analyses for cSMS (Wilcoxon rank-sum test) and RQLQ (Welch t-test) based on subject-level aggregates during peak pollen seasons showed no significant difference (3.9% favoring crossover with p=0.59% and 3.9% favoring allergen with p=0.19, respectively).

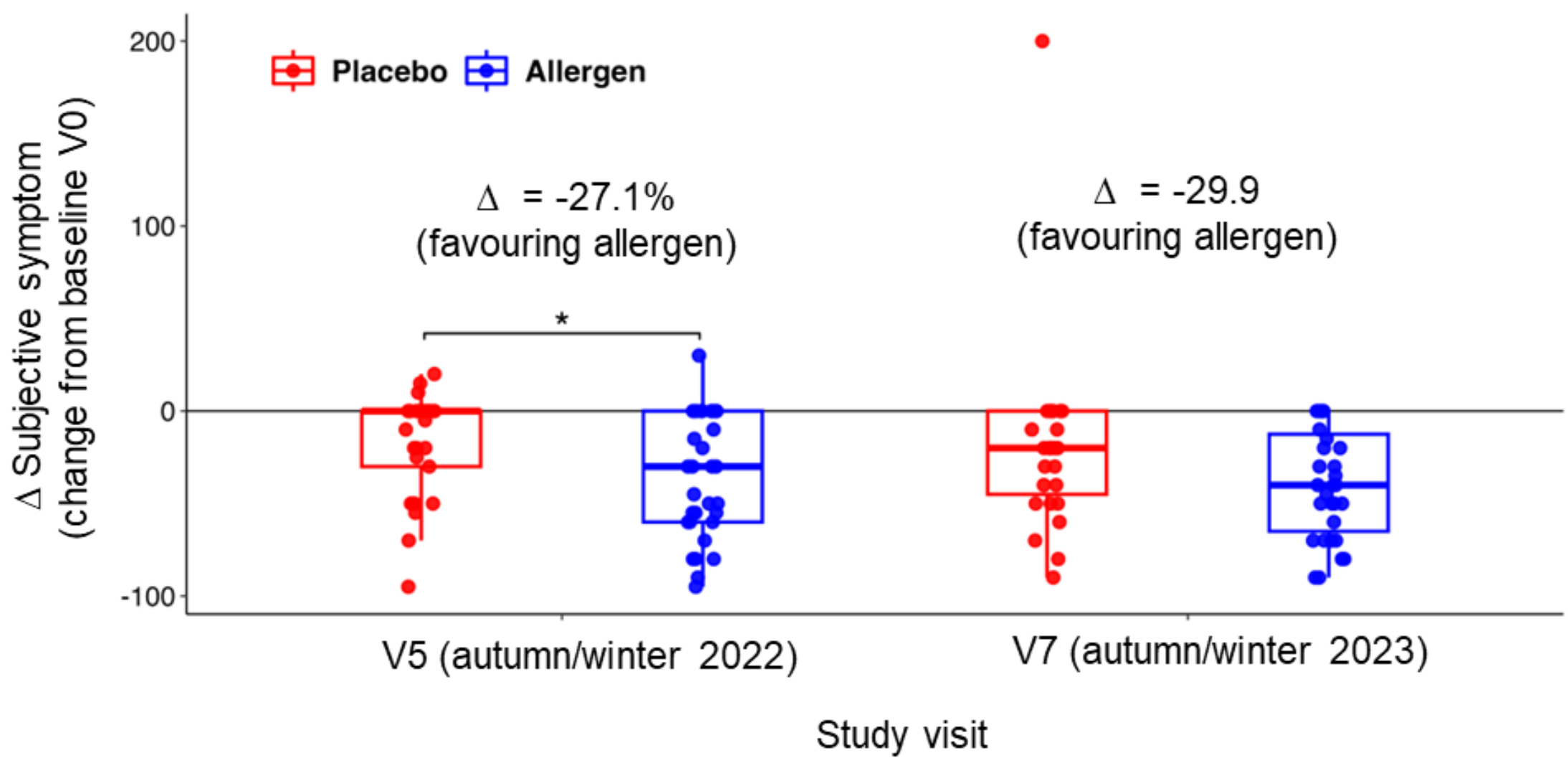

Supplementary Figure S5. Subjective symptom improvement after pollen seasons. Subjective symptom scores were assessed after pollen seasons 2022 (V5) and 2023 (V7). Baseline symptom levels prior to treatment were set to 100%, and changes are shown relative to baseline (Δ); negative values indicate symptom improvement. Boxplots display the median and interquartile range, with whiskers representing 1.5 times the interquartile range and dots indicating individual participants. For between-group comparisons Wilcoxon rank-sum test was used. *$p < 0.05$.

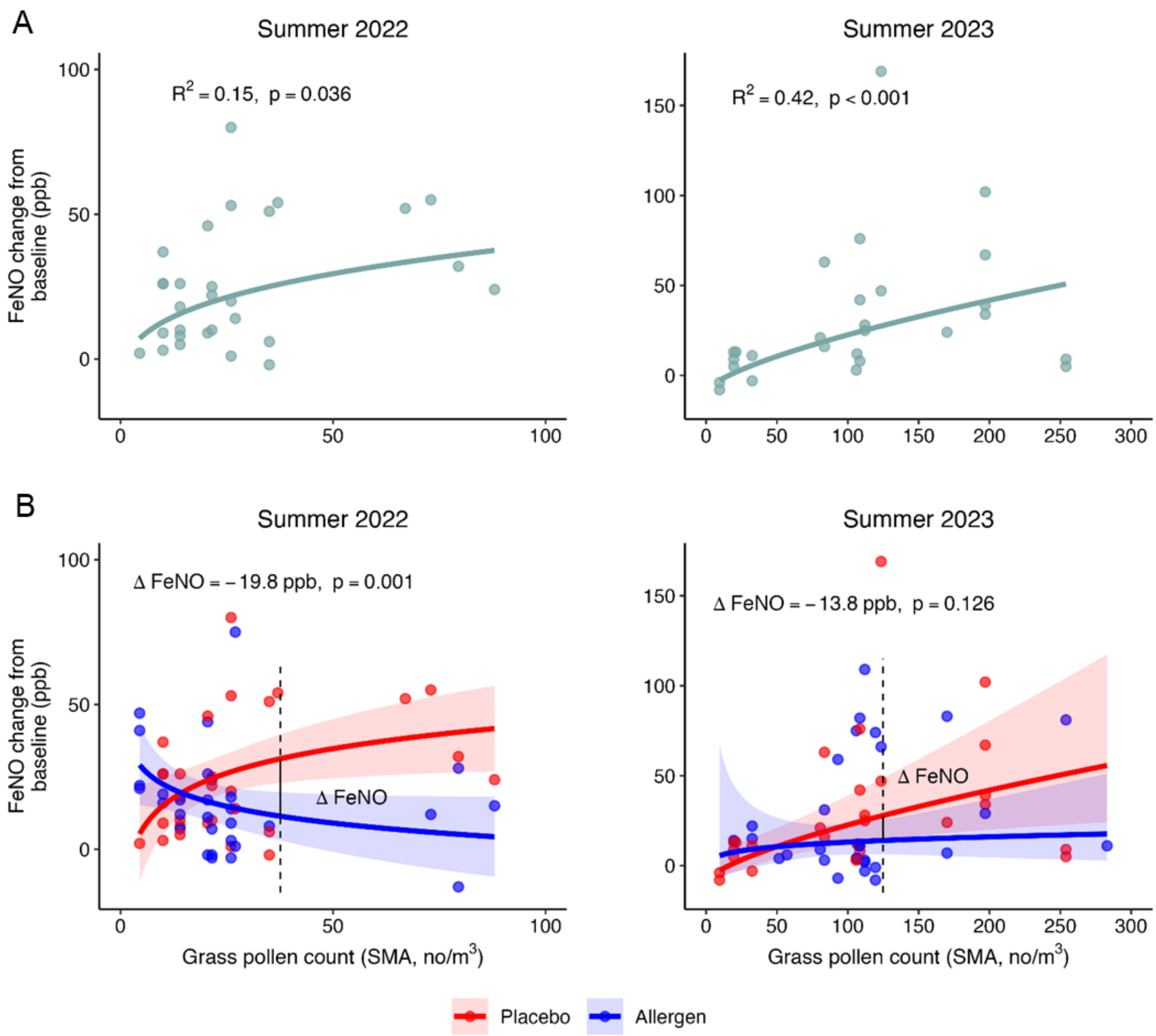

Supplementary Figure S6. Influence of pollen load on ILIT-associated on lung function changes. (A) shows linear regressions between log-transformed simple moving averages (SMA) of grass pollen counts and FeNO change from baseline in the placebo group during summer 2022 (left) and 2023 (right). **(B)** To estimate the effect of pollen load on ILIT efficacy, regression models were extended to include data from the ILIT group and additional covariates for summer 2022 (left) and summer 2023 (right). Dashed lines indicate the mean grass pollen counts during the respective follow-up periods (summer 2022: 37.6 grains/m³; summer 2023: 124.9 grains/m³). ΔFeNO denotes the between-group difference (allergen vs placebo) in FeNO change from baseline at the mean pollen count of the follow-up period. Grey shaded areas represent 95% confidence intervals.

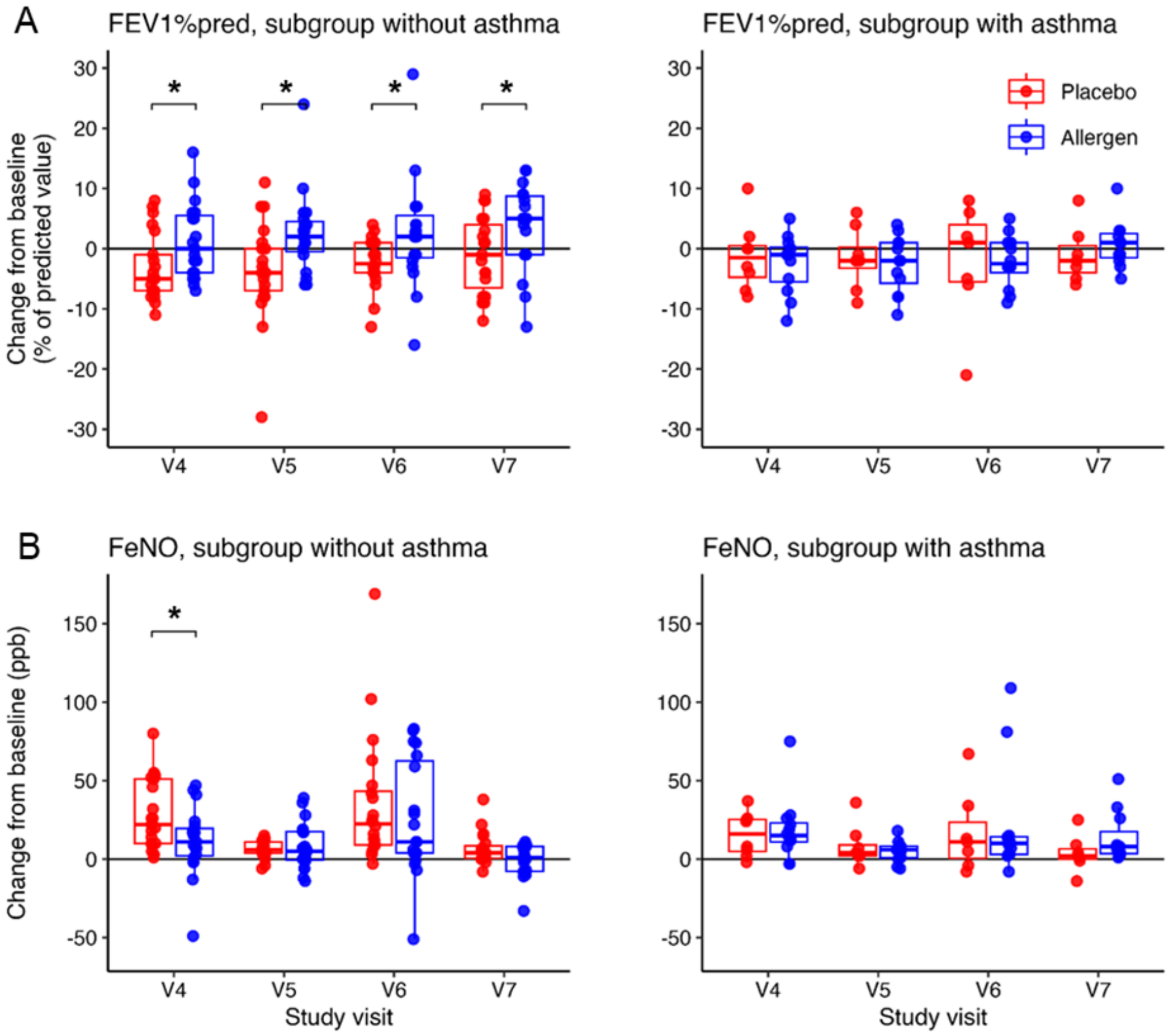

Supplementary Figure S7. Asthma subgroup analysis of ILIT efficacy. (A) Changes in FEV1%pred in non-asthmatic (left) and asthmatic participants (right) and **(B)** changes in FeNO in non-asthmatic (left) and asthmatic participants (right). FEV1%pred denotes FEV1 expressed as percentage of the predicted value. Boxplots show the 25th percentile, median, and 75th percentile; dots represent individual participants. Between-group comparisons were performed using the Mann–Whitney U test. $^*p < 0.05$.
